# Supplementary material for: Time elapsed between Zika and dengue virus infections affects antibody and T cell responses
Source: Nat Commun. 2019 Sep 20;10:4316. doi: 10.1038/s41467-019-12295-2 (PMC6754404; doi:10.1038/s41467-019-12295-2)
Supplement: Supplementary file 1 — Supplementary Information [file 41467_2019_12295_MOESM1_ESM.pdf]

Time elapsed between Zika and dengue virus infections affects antibody and T cell responses

Pérez-Guzmán *et al.*

Supplementary Information

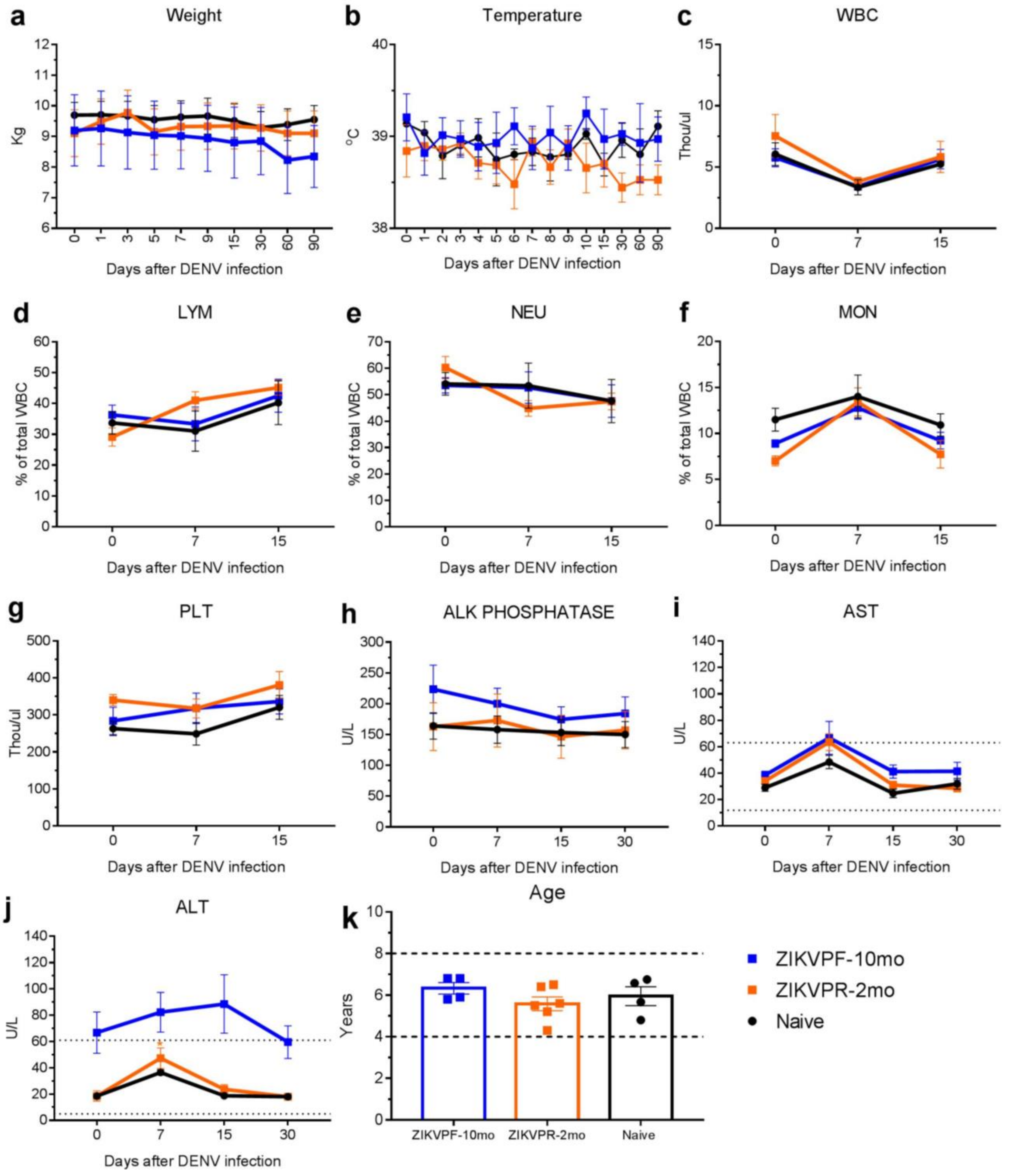

Supplementary Figure 1 | Clinical status and vital signs kinetics in ZIKV-immune and naïve macaques. (**a**) Weight (kg) was measured at baseline, 1, 3, 5, 7, 9, 15, 30, 60 and 90 dpi. (**b**) Temperature (°C) was monitored with an infrared device at baseline, 1-10, 15, 30, 60 and 90 dpi. Complete blood cell counts (CBC) parameters (thou/ul and/or % of total WBC) such as (**c**) white blood cells (WBC), (**d**) lymphocytes (LYM), (**e**) neutrophils (NEU), (**f**) monocytes (MON), and (**g**) platelets (PLT) were screened at baseline, 7, and 15 dpi. Comprehensive metabolic panel (CMP)

was performed to assess levels (U/L) of (**h**) alkaline phosphatase (ALK PHOSPHATASE) and liver enzymes (**i**) aspartate transaminase (AST), and (**j**) alanine transaminase (ALT) at baseline, 7, 15 and 30 dpi. Normal range of AST and ALT are depicted for reference. (**k**) Age of rhesus macaques are depicted including the range of young adults for reference. Symbols represent mean level detected for each parameter per cohort per timepoint: blue squares (ZIKVPF-10mo), orange squares (ZIKVPR-2mo) and black circles (Naïve). Lines connect mean values detected over time. Error bars indicate the standard error of the mean (SEM) for each cohort per timepoint. Statistically significant differences between groups were determined using Two-Way Anova adjusted for Tukey's multiple comparisons test including 10, 15, 3, 4, and 3 families for panel a, b, c-g, h-j, and k, respectively, and 3 comparisons per family. For differences in ALT levels Two-Way Anova Dunnett's multiple comparisons test (comparison of each group response at each timepoint versus baseline of the same group) was performed including 3 families, and 3 comparisons per family due to divergence of non-specific levels between cohorts at baseline. Statistically differences are reported as multiplicity adjusted *p* values (* <0.05).

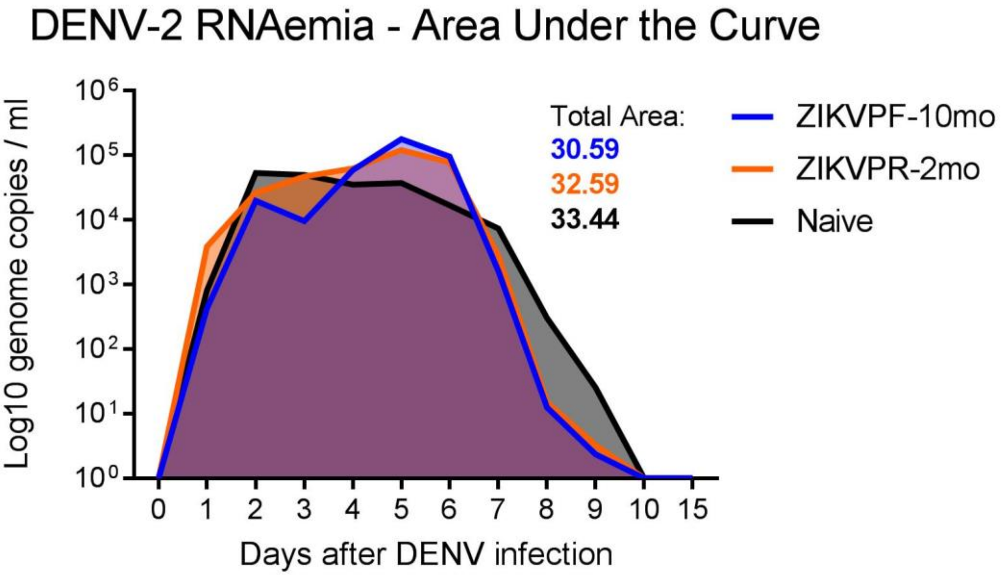

Supplementary Figure 2 | Previous ZIKV immunity modulates DENV RNAemia kinetics and is associated with a lower area under the curve. The area under the curve (AUC) was calculated using log-transformed values of DENV-2 RNAemia in ZIKV-immune and naïve animals. The total area by group is depicted on the graph as light blue, light orange and gray for ZIKVPF-10mo, ZIKVPR-2mo, and Naïve, respectively. Lines mark the mean value of genome copies per group per timepoint. A value of 1 was assigned to all samples below the LOD in order to calculate the means.

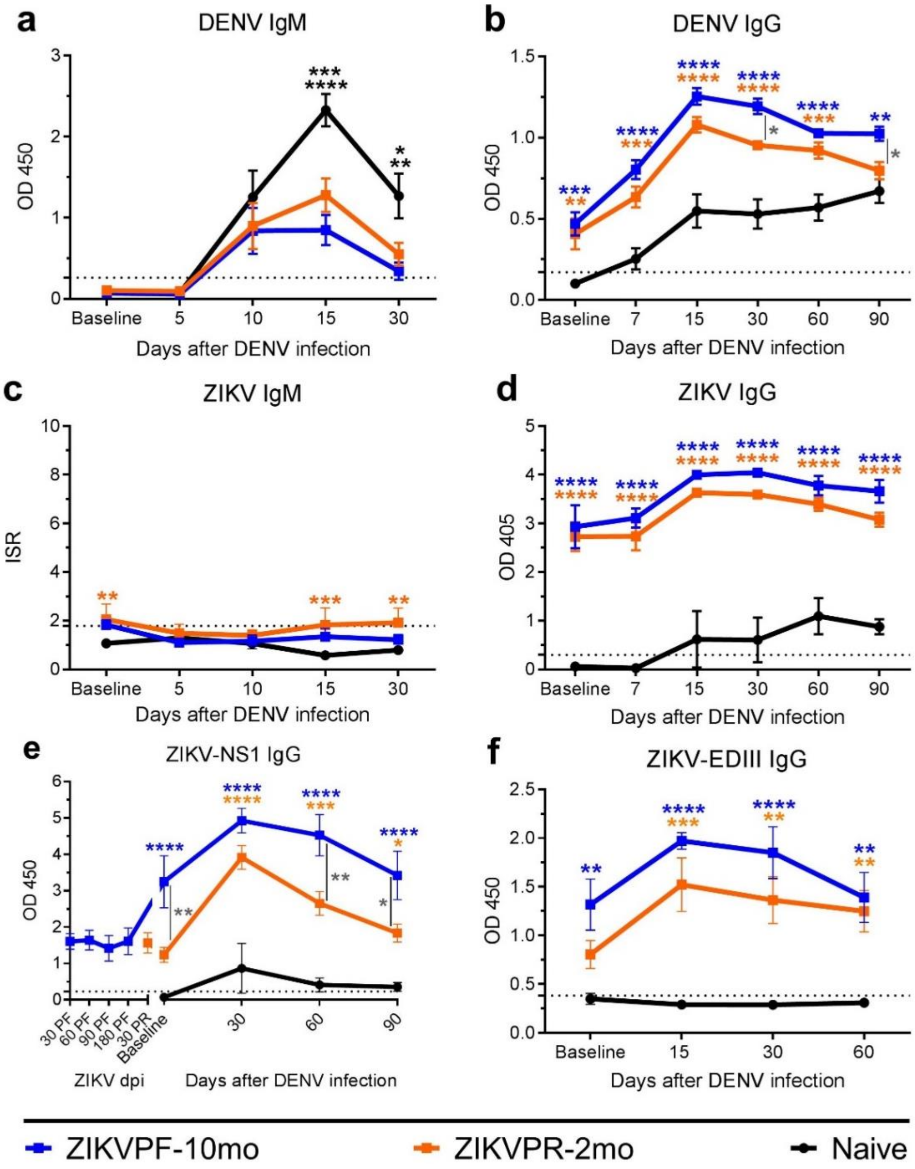

Supplementary Figure 3 | Serological cross-reactivity is boosted by ZIKV immunity. Levels of DENV (**a**) IgM and (**b**) IgG, and ZIKV (**c**) IgM, (**d**) IgG, (**e**) NS1-IgG and (**f**) EDIII-IgG were measured by ELISA at multiple timepoints before and after DENV infection. Symbols connected with full lines represent mean levels of Abs detected per cohort over time: blue squares (ZIKVPF-10mo), orange squares (ZIKVPR-2mo) and black circles (Naïve). Panel e includes additional timepoints before DENV infection for ZIKV-immune groups: 30, 60, 90 and 180 days after ZIKV (H/PF/2013) infection for the ZIKVPF-10mo group, and 30 days after ZIKV (PRVABC59) infection for the ZIKVPR-2mo group. Error bars indicate the standard error of the mean (SEM) and dotted line mark the limit of detection for each individual ELISA. Results were read at OD 450, 405 or using ISR (Immune Status Ratio) following manufacturer's instructions. Statistically significant differences between groups were calculated using Two-Way Anova adjusted for Tukey's multiple comparisons test including 5, 6, 9, and 4 families, and 3 comparisons per family. Significant multiplicity adjusted *p* values (* <0.05, ** <0.01, *** <0.001, **** <0.0001) are shown. Blue and orange asterisks represent significant difference between the corresponded ZIKV immune groups and naive group, and gray asterisks indicate a significant difference between ZIKV immune groups.

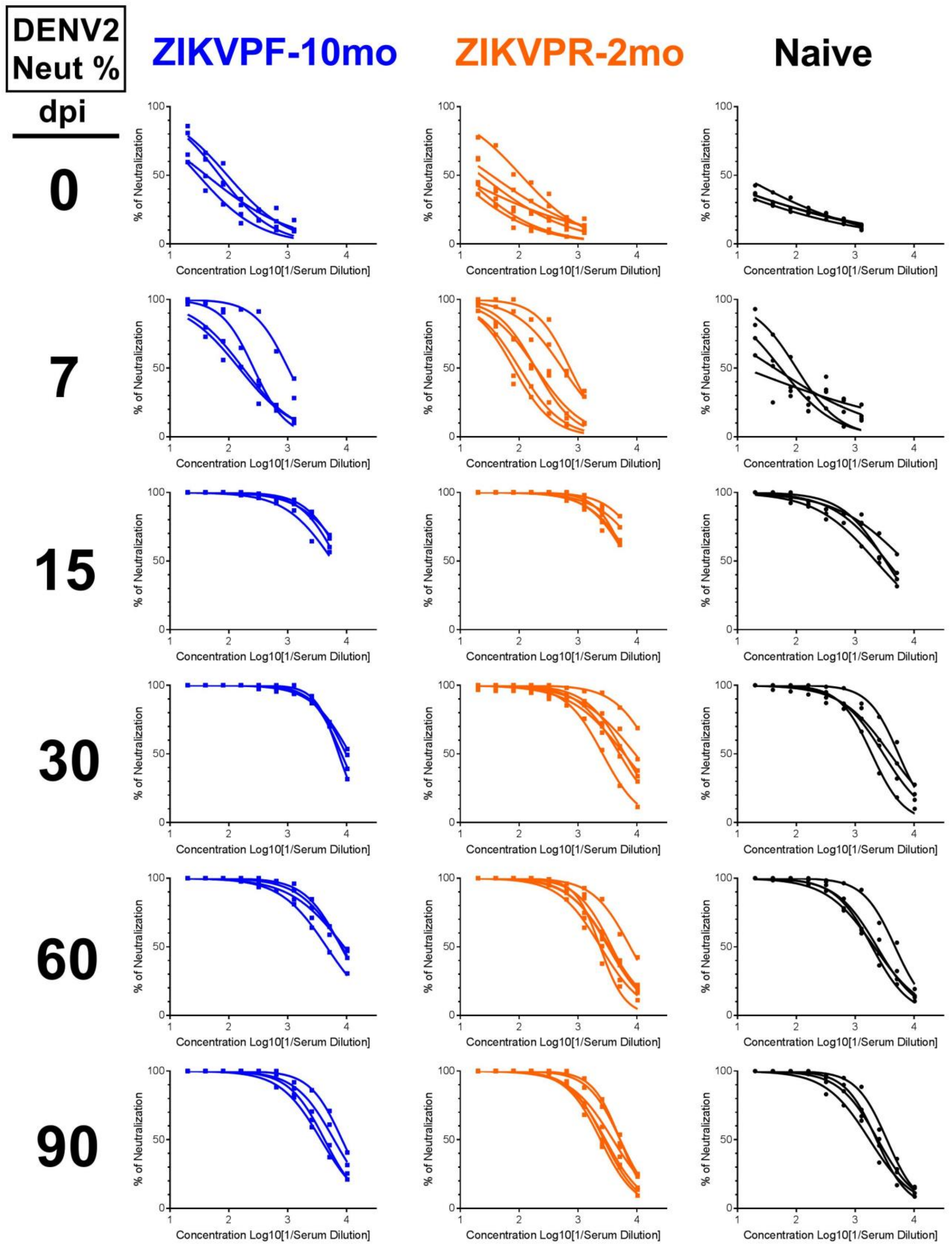

Supplementary Figure 4 | Neutralization kinetics against DENV-2. Percentage of DENV-2 neutralization of each animal per group calculated by the transformation of PRNT60 Neut 2-fold

titers into Log10 (1/serum dilution), and sigmoidal-dose response curves were generated. Each column of panels represent the % of DENV-2 neutralization for each group (ZIKVPF-10mo: blue squares/curves; ZIKVPR-2mo: orange squares/curves; Naïve: black circles/curves) and each row of panels represent a timepoint before and after DENV infection (baseline, 7, 15, 30, 60, 90 dpi).

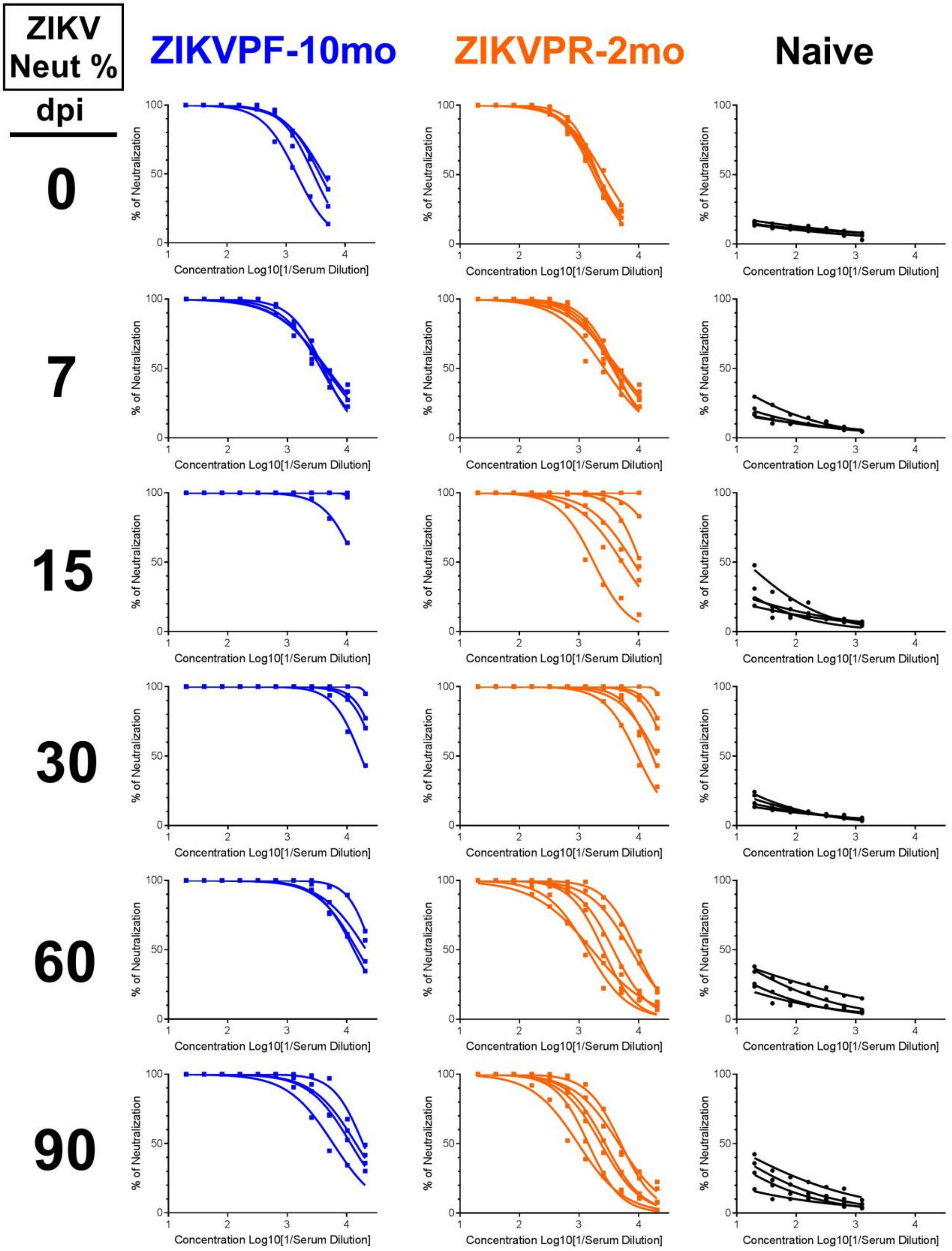

Supplementary Figure 5 | Neutralization kinetics against ZIKV. Percentage of ZIKV (H/PF/2013) neutralization of each animal per group calculated by the transformation of PRNT60

Neut 2-fold titers into Log10 (1/serum dilution), and sigmoidal-dose response curves were generated. Each column of panels represent the % of ZIKV neutralization for each group (ZIKVPF-10mo: blue squares/curves; ZIKVPR-2mo: orange squares/curves; Naïve: black circles/curves) and each row of panels represent a timepoint before and after DENV infection (baseline, 7, 15, 30, 60, 90 dpi).

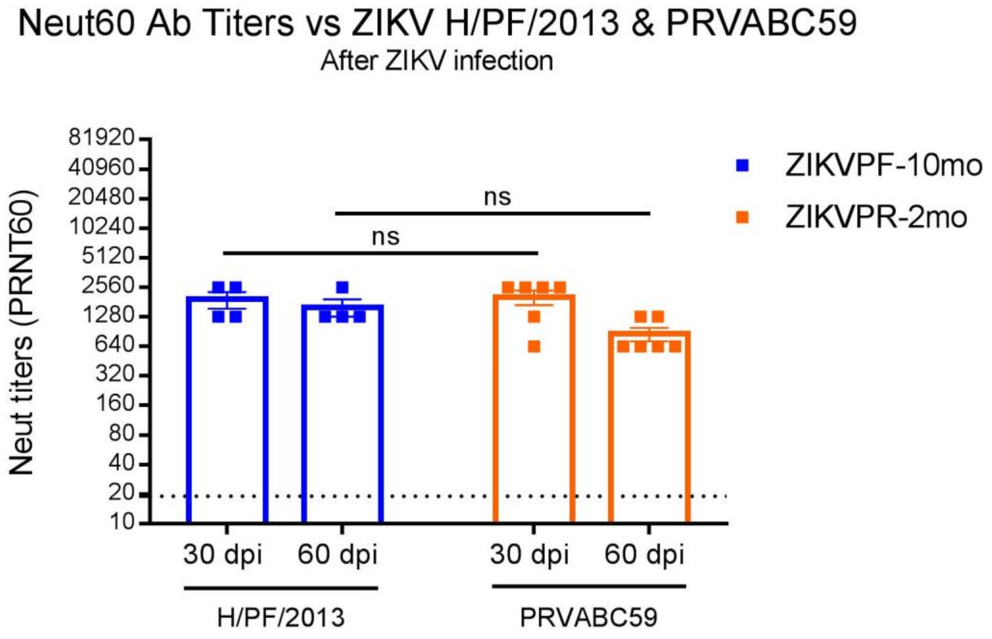

Supplementary Figure 6 | Similar neutralizing titers induced by two different ZIKV strains. NAb titers against H/PF/2013 and PRVABC59 ZIKV strains for ZIKVPF-10mo and ZIKVPR-2mo groups, respectively, were determined by PRNT60 at 30 and 60 after ZIKV infection. Symbols indicate levels of NAb titers detected per animal: blue squares (ZIKVPF-10mo), and orange squares (ZIKVPR-2mo). Error bars represent the standard error of the mean (SEM). PRNT60: NAb titer capable of reduce 60% or more of ZIKV strains plaque-forming units (pfu) compared with the mock (control of virus without serum). A PRNT60 1:20 titer was considered positive, and <1:20 as a negative Neut titer. Dotted line mark <1:20 for negative results. Statistically significant differences (ns: not significant) between two groups were calculated using Two-Way Anova corrected for Sidak's multiple comparisons test including 1 family, and 2 comparisons within the family.

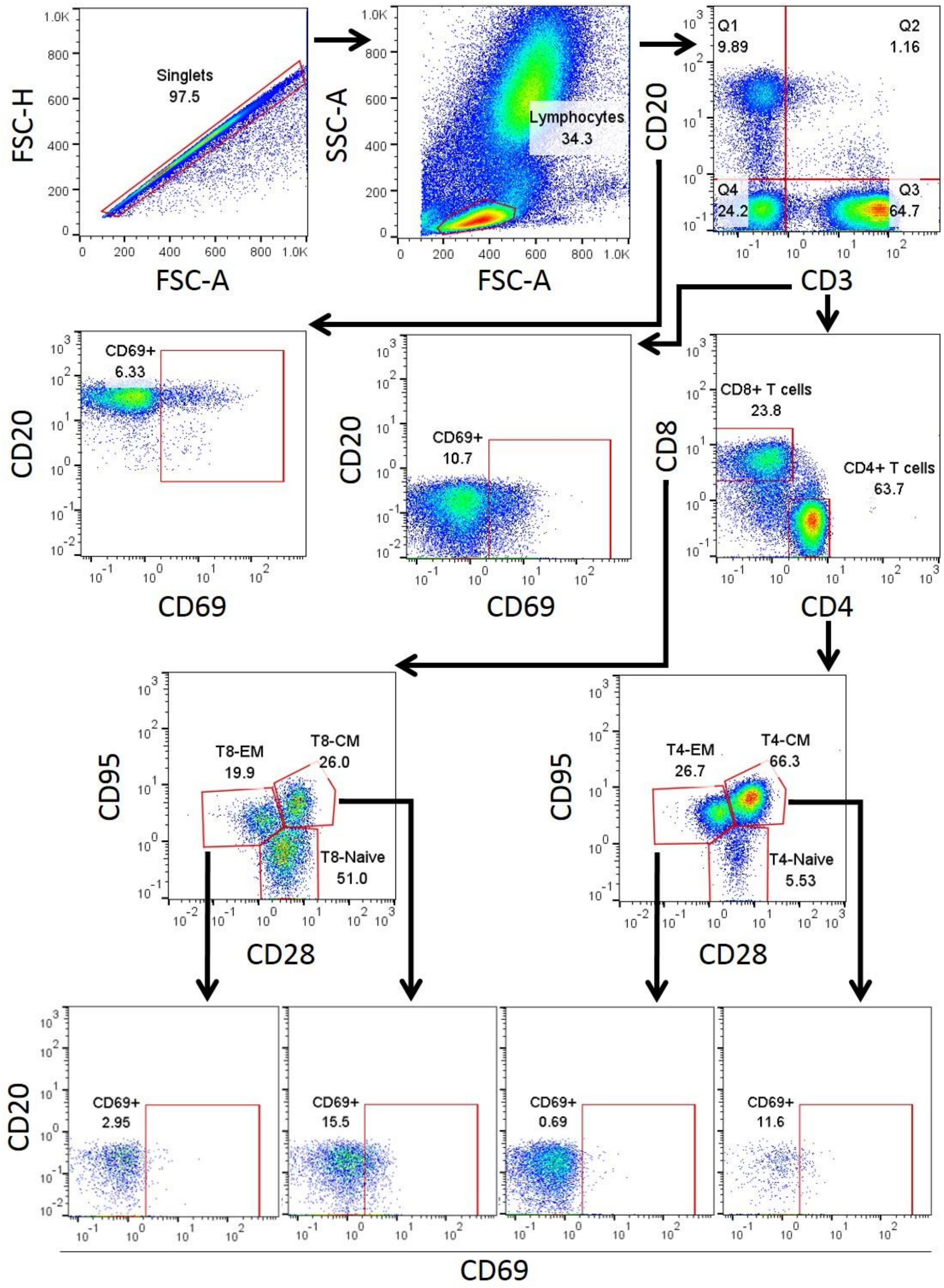

Supplementary Figure 7 | Gating strategy for immunophenotyping and activation of B cells, and memory T cell subpopulations. Single cells (singlets) were selected by their FSC area

(FSC-A) and height (FSC-H) patterns. Lymphocytes (LYM) were gated based on their characteristic forward and side scatter pattern (FSC, SSC). T cells were selected gating on the $CD3^+$ population. $CD4^+$ and $CD8^+$ T cells were defined as $CD3^+CD4^+$ and $CD3^+CD8^+$, respectively. Naive (N; $CD28^+CD95^-$), effector memory (EM; $CD28^-CD95^+$) and central memory (CM; $CD28^+CD95^+$) T cell subpopulations were determined within $CD4^+$ and $CD8^+$ T cells. B cells were defined as $CD20^+CD3^-$. The activation of B and T cell memory subpopulations (EM and CM) was assessed by the presence of the early activation marker CD69.

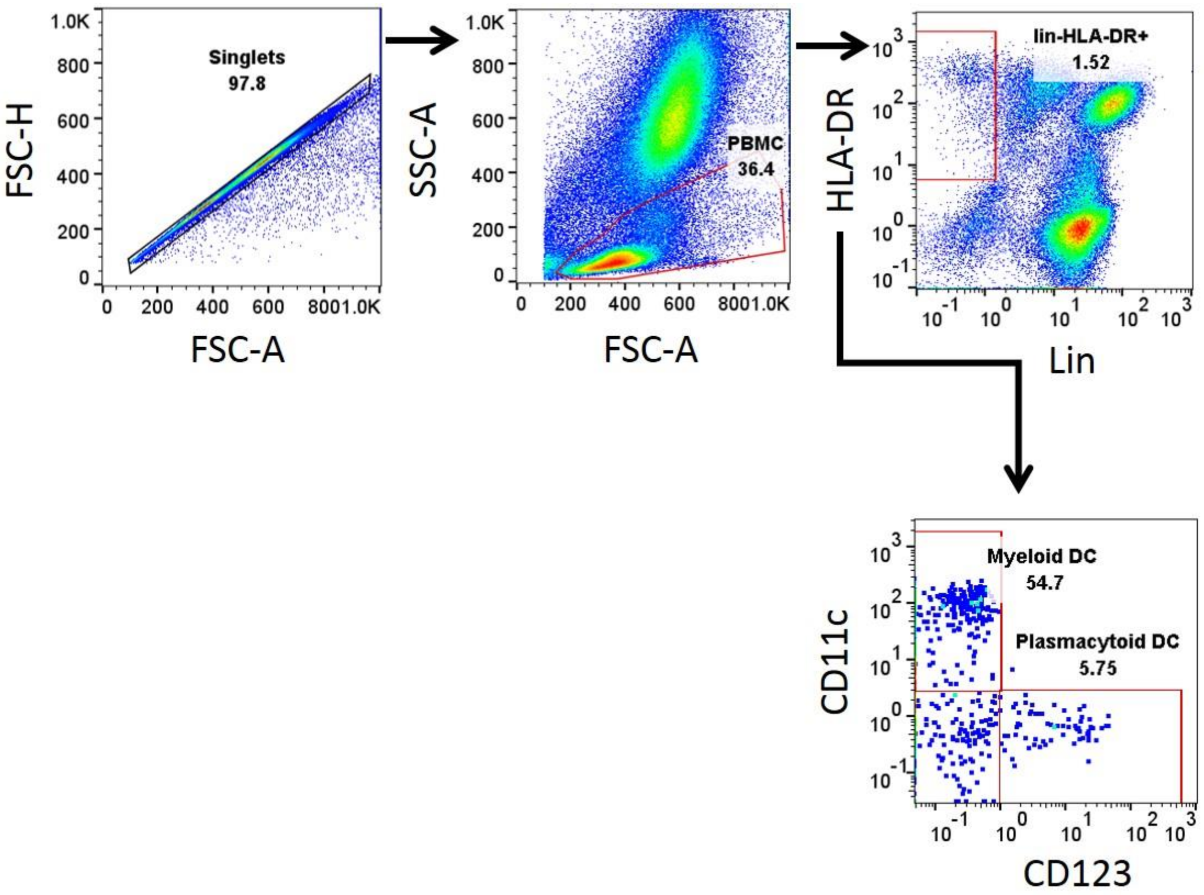

Supplementary Figure 8 | Gating strategy for immunophenotyping of plasmacytoid and myeloid dendritic cells. Single cells (singlets) were selected by their FSC area (FSC-A) and height (FSC-H) patterns. Lymphocytes (LYM) were gated based on their characteristic forward and side scatter pattern (FSC, SSC). Dendritic cells (DC) were separated in two populations within the Lineage-DR+ (HLA-DR$^+$ CD3$^-$ CD14$^-$ CD16$^-$ CD20$^-$ CD8$^-$ NKG2A$^-$) by the expression of CD123 (plasmacytoid, pDC) or CD11c (myeloid, mDCs).

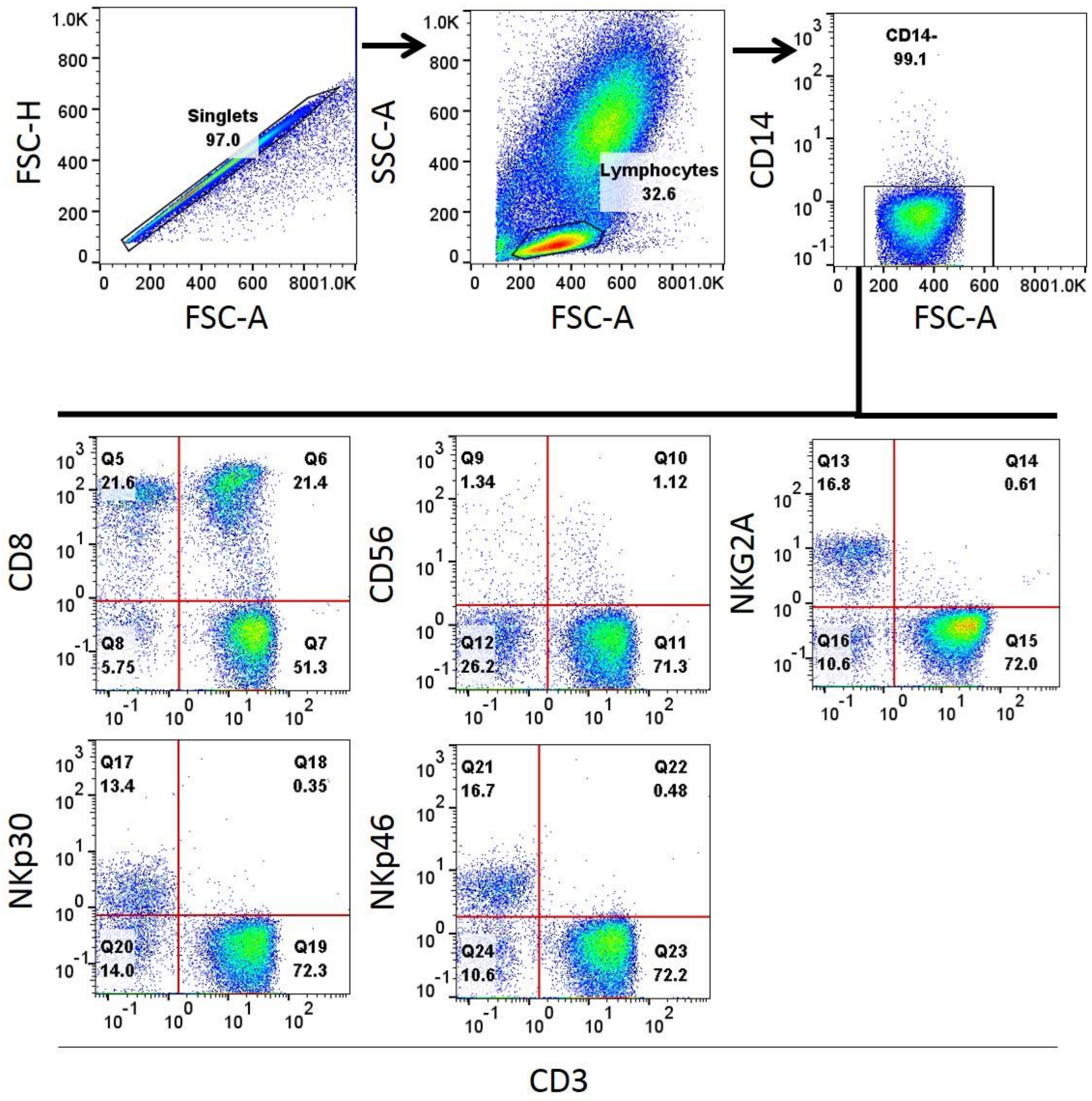

Supplementary Figure 9 | Gating strategy for Natural killer cell subpopulations. Single cells (singlets) were selected by their FSC area (FSC-A) and height (FSC-H) patterns. Lymphocytes (LYM) were gated based on their characteristic forward and side scatter pattern (FSC, SSC). Natural killer (NK) cells were defined as $CD3^{-}CD20^{-}CD14^{-}$ and analyzed by the double positive expression of the following NK cell markers: CD8, CD56, NKG2A, NKp30, and NKp46.

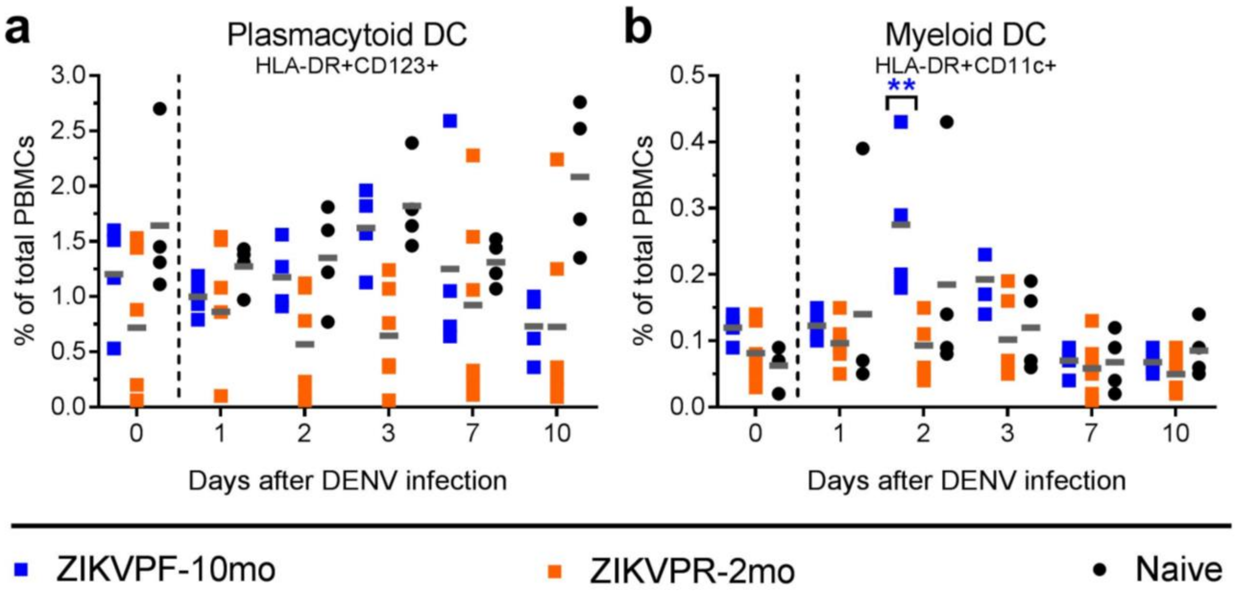

Supplementary Figure 10 | Dendritic cells subsets modulation induced by DENV infection. The frequency (% of total PBMCs) of dendritic cells (DCs) subsets including (**a**) plasmacytoid (pDCs: Lin-HLA-DR^{+}CD123^{+}) and (**b**) myeloid (mDCs: Lin-HLA-DR^{+}CD11c^{+}) was assessed before and up to 10 days after DENV infection by immunophenotyping using flow cytometry analysis. Symbols represent individual animals per group for each timepoint: blue squares (ZIKVPF-10mo), orange squares (ZIKVPR-2mo) and black circles (Naïve). Short gray lines depict mean value for each group detected overtime. Cutted line divide % of DCs quantified before and after DENV infection. Statistically significant differences within groups were determined using Two-Way Anova Dunnett's multiple comparisons test (comparison of each group response at each timepoint versus baseline of the same group) including 3 families, and 5 comparisons per family. Significant differences are reported as multiplicity adjusted *p* values (* <0.05, ** <0.01, *** <0.001, **** <0.0001). Asterisks represent significant difference between the corresponded timepoint and baseline within the same group.

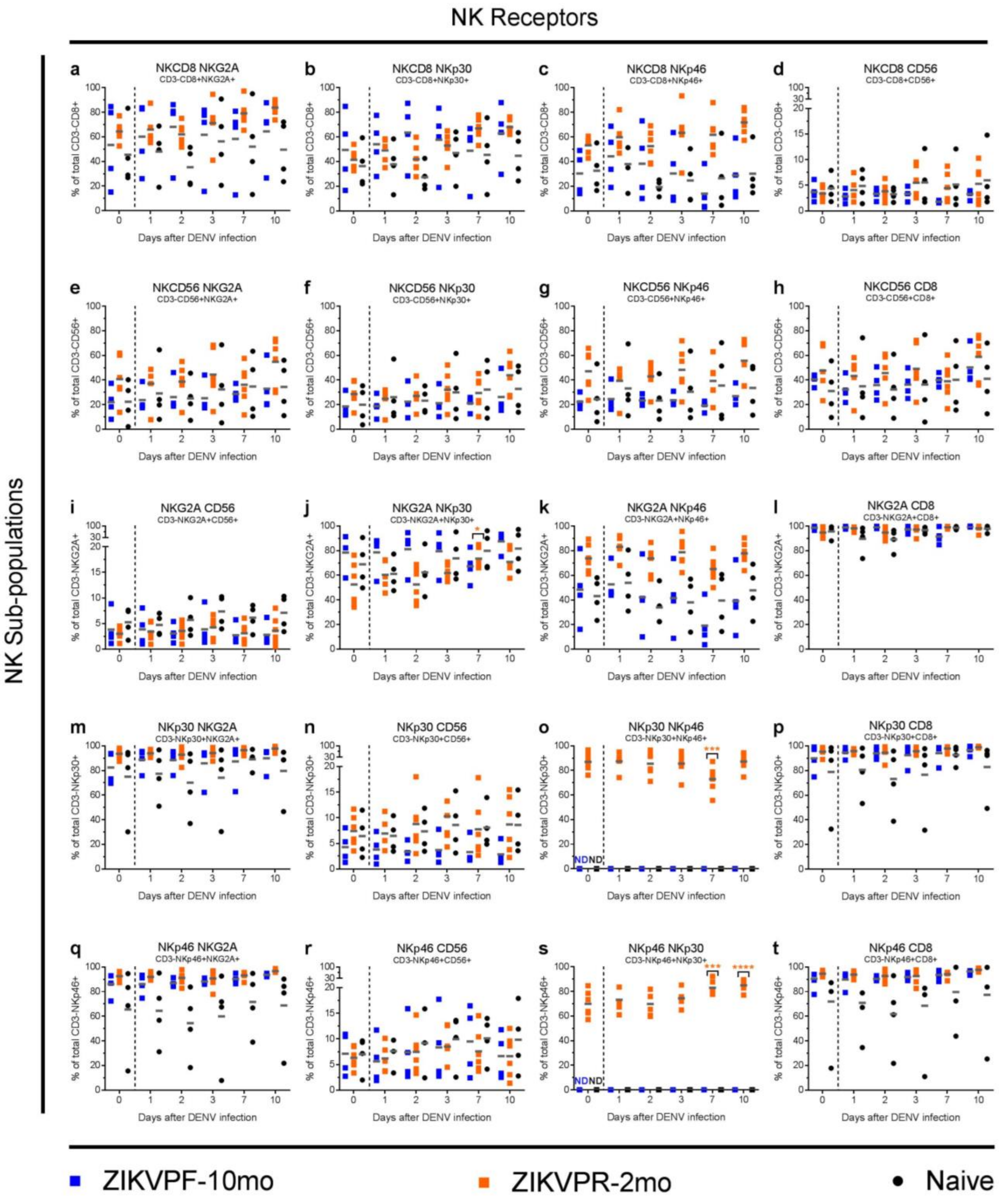

Supplementary Figure 11 | Natural killer cell subpopulations and their differential receptors expression. Natural killer (NK) cell subpopulations and the relative expression of multiple NK receptors within each subpopulation: (**a-d**) NKCD8, (**e-h**) NKCD56, (**i-l**) NKG2A, (**m-p**) NKp30 and (**q-t**) NKp46 were quantified by immunophenotyping using flow cytometry analysis before and up to 10 days after DENV infection. Individual symbols represent each animal per group over

time: blue squares (ZIKVPF-10mo), orange squares (ZIKVPR-2mo) and black circles (Naïve). Short gray lines mark mean value for each group. Cutted line divide % of NK cells quantified before and after DENV infection. Statistically significant differences within groups were determined using Two-Way Anova Dunnett's multiple comparisons test (comparison of each group response at each timepoint versus baseline of the same group) including 3 families, and 5 comparisons per family. Significant differences are reported as multiplicity adjusted *p* values (* <0.05, ** <0.01, *** <0.001, **** <0.0001). Asterisks represent significant difference between the corresponded timepoint and baseline within the same group. ND (Not Done) in panels *8o* and *8s* refers that for ZIKVPF-10mo and Naïve groups the $NKp30^{+}NKp46^{+}$ and $NKp46^{+}NKp30^{+}$ subpopulations were not measured.

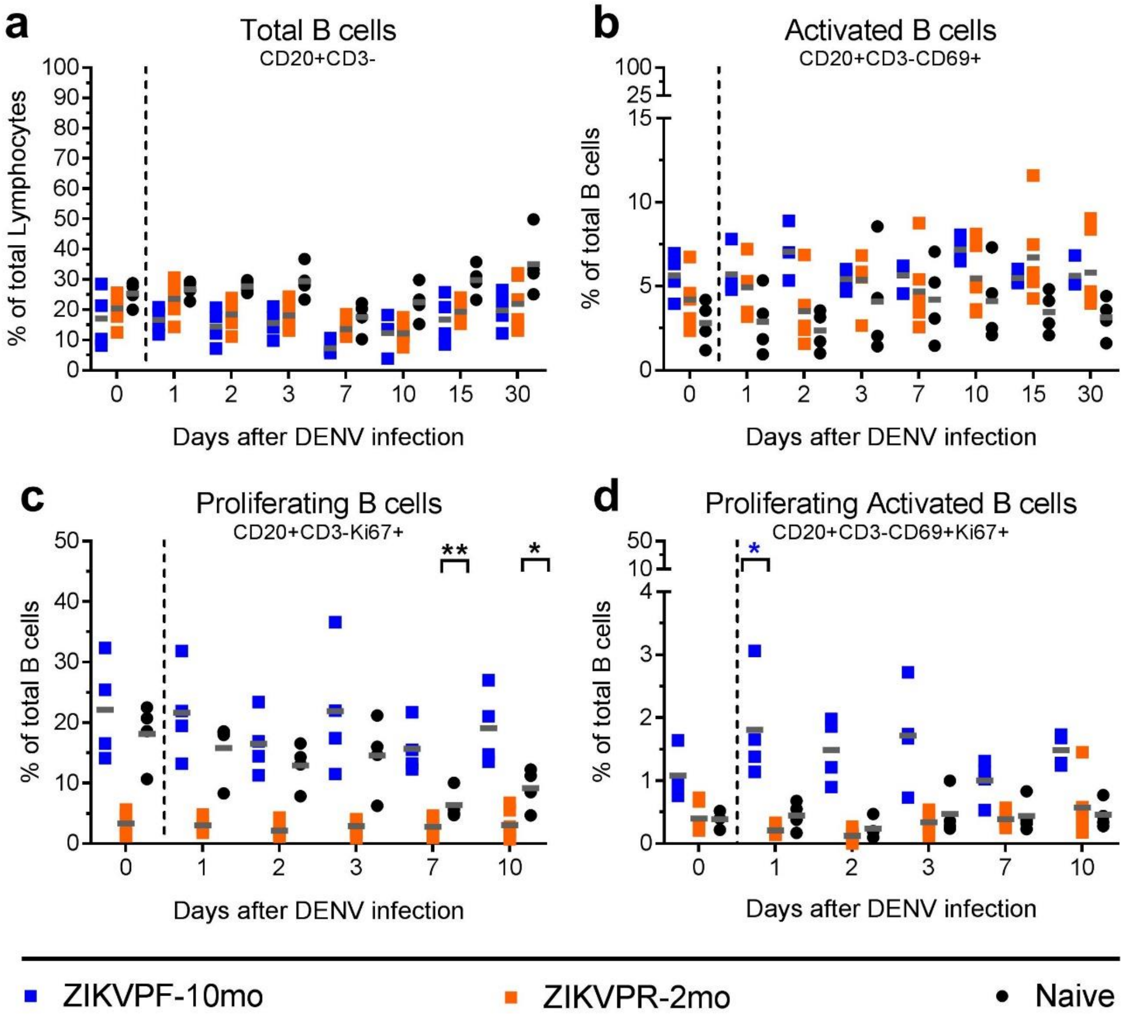

Supplementary Figure 12 | B cells proliferation and activation higher in ZIKV middle-convalescent macaques. The (**a**) total (% of total Lymphocytes), (**b**) activated, (**c**) proliferating, and (**d**) proliferating/activated B cells (% of total B cells) were determined at baseline and following DENV infection by immunophenotyping using flow cytometry analysis. B cells proliferation and activation were monitored since baseline up to 10 and 30 dpi, respectively. Symbols represent individual animals per group for each timepoint: blue squares (ZIKVPF-10mo), orange squares (ZIKVPR-2mo) and black circles (Naïve). Short gray lines depict mean value of B cells percent in each group of animals per timepoint. Cutted line divide % of B cells quantified before and after DENV infection. Statistically significant differences within groups were determined using Two-Way Anova Dunnett's multiple comparisons test (comparison of each group response at each timepoint versus baseline of the same group) including 3 families, and 7 and 5 comparisons per family in panels a-b and c-d, respectively. Significant differences are reported as multiplicity adjusted *p* values (* <0.05, ** <0.01). Asterisks represent significant difference between the corresponded timepoint and baseline within the same group.

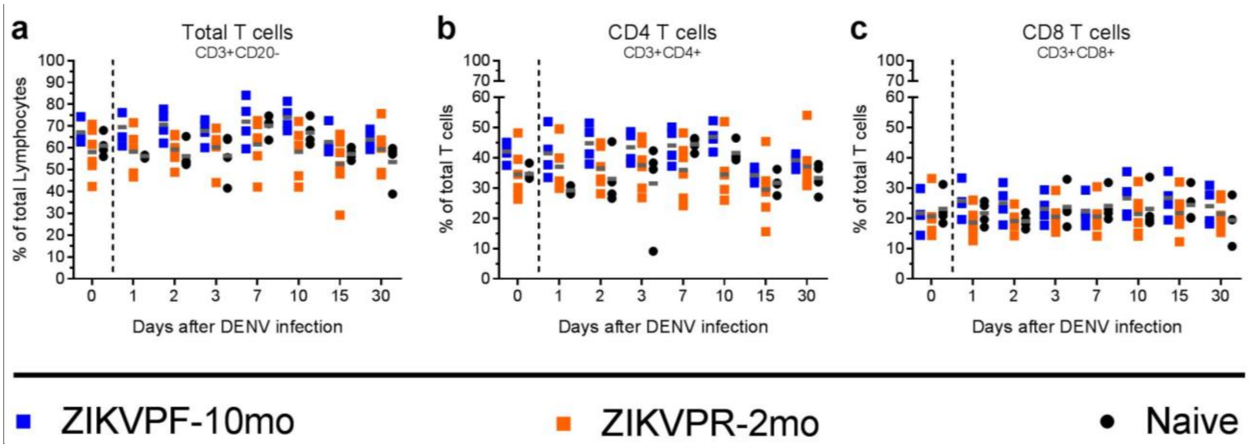

Supplementary Figure 13 | Comparable T cells frequency between groups. The (**a**) total T cells (% of total Lymphocytes), (**b**) CD4$^+$ and (**c**) CD8$^+$ T cell compartments (% of total T cells) frequencies were quantified at baseline and following DENV infection up to 30 dpi by immunophenotyping using flow cytometry. Symbols represent individual animals per group for each timepoint: blue squares (ZIKVPF-10mo), orange squares (ZIKVPR-2mo) and black circles (Naïve). Short gray lines mark mean value of T cells percent in each cohort per timepoint. Cutted line divide % of T cells quantified before and after DENV infection. Statistically significant differences within groups were determined using Two-Way Anova Dunnett's multiple comparisons test (comparison of each group response at each timepoint versus baseline of the same group) including 3 families, and 7 comparisons per family.

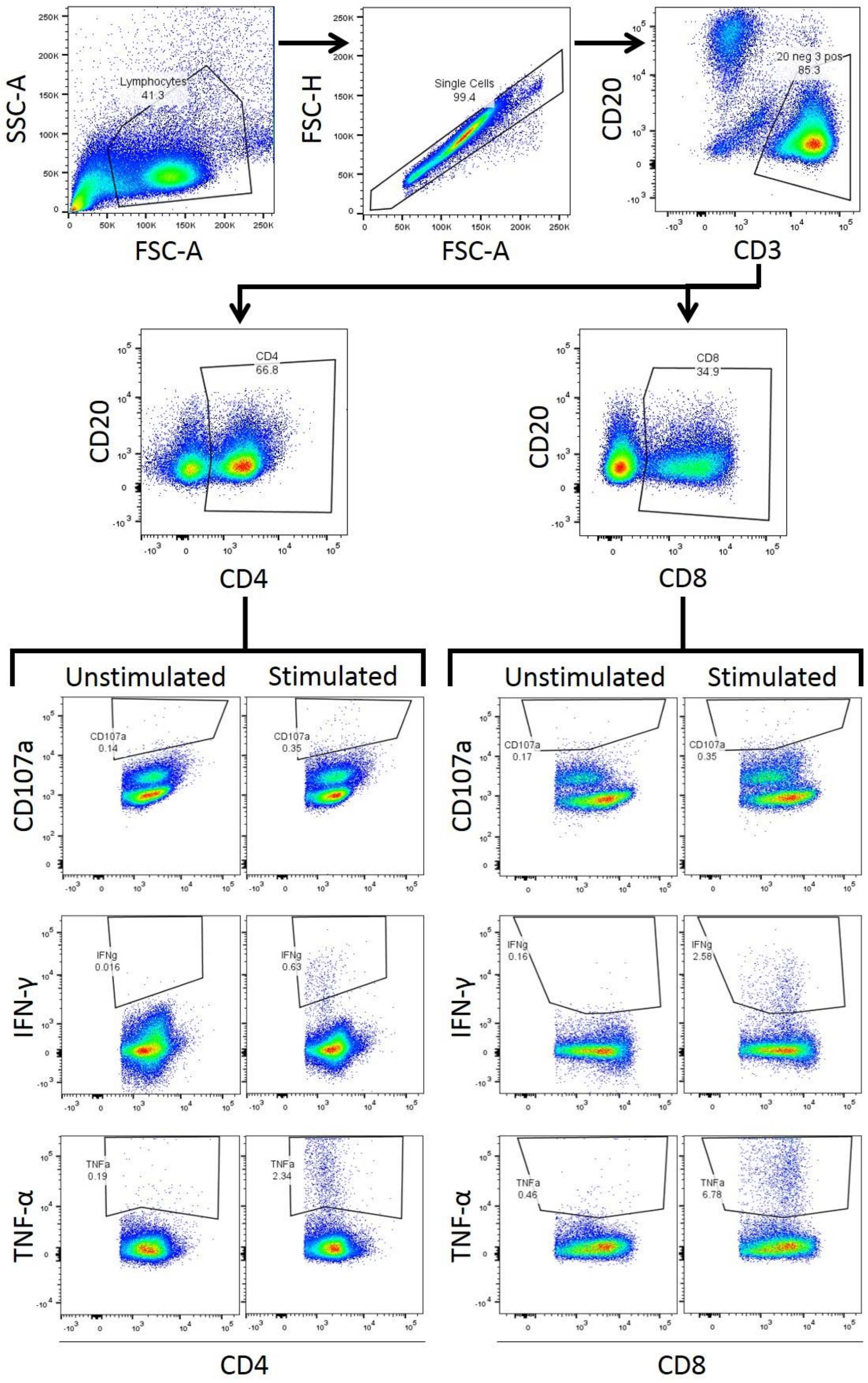
SSC-A
FSC-A
Lymphocytes
41.3
FSC-H
Single Cells
99.4
CD20
CD3
20 neg 3 pos
85.3
CD4
66.8
CD8
34.9
Unstimulated
Stimulated
CD107a
0.14
0.35
0.17
IFN-γ
IFNg
0.016
0.63
0.16
2.58
TNF-α
TNFa
0.19
2.34
0.46
6.78

Supplementary Figure 14 | Gating strategy for CD4+ and CD8+ T cell functional response. After stimulation, lymphocytes (LYM) were gated based on their characteristic forward and side scatter pattern (FSC, SSC). Single cells (singlets) were selected by their FSC area (FSC-A) and height (FSC-H) patterns. Cells were stained for the following markers: CD3, CD4, CD8, CD20 (excluded), CD107a (functional cytotoxicity). Levels of IFN-γ and TNF-α also were measured in gated lymphocytes cell populations.

Supplementary Table 1 | Sequence alignment and amino acid identity of ZIKV strains PRVABC59 and H/PF/2013.

Pairwise alignment of both ZIKV strains sequences.<br>(Sequences downloaded from ViPR database and global alignment was performed using Blosum62 in Genious Software).	>99.99% amino acid identity
Envelope (E) protein region of both ZIKV strains.	Identical
Amino acids residues changes between both ZIKV strains. Marked in red within sequences.<br><br>From ZIKV-PR → ZIKV-PF	T_{80}→ I (Capsid)<br>G_{892}→W (NS1)<br>V_{2611}→A (NS5)<br>V_{2634}→M (NS5)
ZIKV-PRVABC59<br><br>Accession number: KX377337	MKNPKKKSGGFRIVNMLKRGVARVSPFGGLKRLPAGLLLGHGPIRMVLAI<br>LAFLRFTAIKPSLGLINRWGSVGKKEAMETIKKFKKDLAAMLRIINARKE<br>KKRRGADTSVGIVGLLLTTAMAAEVTRRGSAYYMYLDRNDAGEAISFPTT<br>LGMNKCYIQIMDLGHMCDATMSYECPMLDEGVEPDDVDCWCNTTSTWVVY<br>GTCHHKKGEARRSRRAVTLPSHSTRKLQTRSQTWLESREYTKHLIRVENW<br>IFRNPGFALAAAAIAWLLGSSTSQKVIYLVMILLIAPAYSIRCIGVSNRD<br>FVEGMSGGTWVDVVLEHGGCVTVMAQDKPTVDIELVTTTVSNMAEVRSYC<br>YEASISDMASDSRCPTQGEAYLDKQSDTQYVCKRTLVDRGWGNGCGLFGK<br>GSLVTCAKFACSKKMTGKSIQPENLEYRIMLSVHGSQHSGMIVNDTGHET<br>DENRAKVEITPNSPRAEATLGGFGSLGLDCEPRTGLDFSDLYYLTMNNKH<br>WLVHKEWFHDIPLPWHAGADTGTPHWNNKEALVEFKDAHAKRQTVVVLGS<br>QEGAVHTALAGALEAEMDGAKGRLSSGHLKCRLKMDKLRLKGVSYSLCTA<br>AFTFTKIPAETLHGTVTVEVQYAGTDGPCKVPAQMAVDMQTLTPVGRLIT<br>ANPVITESTENSKMMLELDPPFGDSYIVIGVGEKKITHHWHRSGSTIGKA<br>FEATVRGAKRMAVLGDTAWDFGSVGGALNSLGKGIHQIFGAAFKSLFGGM<br>SWFSQILIGTLLMWLGLNTKNGSISLMCLALGGVLIFLSTAVSADVGCSV<br>DFSKKETRCGTGVFVYNDVEAWRDRYKYHPDSPRRLAAAVKQAWEDGICG<br>ISSVSRMENIMWRSVEGELNAILEENGVQLTVVVGSVKNPMGRGPQRLPV<br>PVNELPHGWKAWGKSYFVRAAKTNNSFVVDGDTLKECPLKHRAWNSFLVE<br>DHGFGVFHTSVWLKVREDYSLECDPAVIGTAVKGKEAVHSDLGYWIESEK<br>NDTWRLKRAHLIEMKTCEWPKSHTLWTDGIEESDLIIPKSLAGPLSHHNT<br>REGYRTQMKGPWHSEELEIRFEECPGTKVHVEETCGTRGPSLRSTTASGR<br>VIEEWCCRECTMPPLSFRAKDGCWYGMEIRPRKEPESNLVRSMVTAGSTD<br>HMDHFSLGVLVILLMVQEGLKKRMTTKIIISTSMAVLVAMILGGFSMSDL<br>AKLAILMGATFAEMNTGGDVAHLALIAAFKVRPALLVSFIFRANWTPRES<br>MLLALASCLLQTAISALEGDLMVLINGFALAWLAIRAMVVPRTDNITLAI<br>LAALTPLARGTLLVAWRAGLATCGGFMLLSLKGKGSVKKNLPFVMALGLT<br>AVRLVDPINVVGLLLLTRSGKRSWPPSEVLTAVGLICALAGGFAKADIEM<br>AGPMAAVGLLIVSYVVSGKSVDMYIERAGDITWEKDAEVTGNSPRLDVAL

	DESGDFSLVEDDGPPMREIILKVVLMTICGMNPIAIPFAAGAWYVYVKTG<br>KRSGALWDVPAPKEVKKGETTDGVYRVMTRRLLGSTQVGVGVMQEGVFHT<br>MWHVTKGSALRSGEGRLDPYWGDVKQDLVSYCGPWKLDAAWDGHSEVQLL<br>AVPPGERARNIQTLPGIFKTKDGDIGAVALDYPAGTSGSPILDKCGRVIG<br>LYGNGVVIKNGSYVSAITQGRREEETPVECFEPSMLKKKQLTVLDLHPGA<br>GKTRRVLPEIVREAIKTRLRTVILAPTRVVAAEMEEALRGLPVRYMTTAV<br>NVTHSGTEIVDLMCHATFTSRLLQPIRVPNYNLYIMDEAHFTDPSSIAAR<br>GYISTRVEMGEAAAIFMTATPPGTRDAFPDSNSPIMDTEVEVPERAWSSG<br>FDWVTDHSGKTVWFVPSVRNGNEIAACLTKAGKRVIQLSRKTFETEFQKT<br>KHQEWDFVVTTDISEMGANFKADRVIDSRRCLKPVILDGERVILAGPMPV<br>THASAAQRRGRIGRNPNKPGDEYLYGGGCAETDEDHAHWLEARMLLDNIY<br>LQDGLIASLYRPEADKVAAIEGEFKLRTEQRKTFVELMKRGDLPVWLAYQ<br>VASAGITYTDRRWCFDGTTNNTIMEDSVPAEVWTRHGEKRVLKPRWMDAR<br>VCSDHAALKSFKEFAAGKRGAAFGVMEALGTLPGHMTERFQEAIDNLAVL<br>MRAETGSRPYKAAAAQLPETLETIMLLGLLGTVSLGIFFVLMRNKGIGKM<br>GFGMVTLGASAWLMWLSEIEPARIACVLIVVFLLLVVLIPEPEKQRSPQD<br>NQMAIIIMVAVGLLGLITANELGWLERTKSDLSHLMGRREEGATIGFSMD<br>IDLRPASAWAIYAALTTFITPAVQHAVTTSYNNYSLMAMATQAGVLFGMG<br>KGMPFYAWDFGVPLLMIGCYSQLTPLTLIVAIILLVAHYMYLIPGLQAAA<br>ARAAQKRTAAGIMKNPVVDGIVVTDIDTMTIDPQVEKKMGQVLLIAVAVS<br>SAILSRTAWGWGEAGALITAATSTLWEGSPNKYWNSSTATSLCNIFRGSY<br>LAGASLIYTVTRNAGLVKRRGGGTGETLGEKWKARLNQMSALEFYSYKKS<br>GITEVCREEARRALKDGVATGGHAVSRGSAKLRWLVERGYLQPYGKVIDL<br>GCGRGGWSYY**V**ATIRKVQEVKGYTKGGPGHEEP**V**LVQSYGWNIVRLKSGV<br>DVFHMAAEPCDTLLCDIGESSSSPEVEEARTLRVLSMVGDWLEKRPGAFC<br>IKVLCPYTSTMMETLERLQRRYGGGLVRVPLSRNSTHEMYWVSGAKSNTI<br>KSVSTTSQLLLGRMDGPRRPVKYEEDVNLGSGTRAVVSCAEAPNMKIIGN<br>RIERIRSEHAETWFFDENHPYRTWAYHGSYEAPTQGSASSLINGVVRLLS<br>KPWDVVTGVTGIAMTDTTPYGQQRVFKEKVDTRVPDPQEGTRQVMSMVSS<br>WLWKELGKHKRPRVCTKEEFINKVRSNAALGAIFEEEKEWKTAVEAVNDP<br>RFWALVDKEREHHLRGECQSCVYNMMGKREKKQGEFGKAKGSRAIWYMWL<br>GARFLEFEALGFLNEDHWMGRENSGGGVEGLGLQRLGYVLEEMSRIPGGR<br>MYADDTAGWDTRISRFDLENEALITNQMEKGHRALALAIIKYTYQNKVVK<br>VLRPAEKGKTVMDIISRQDQRGSGQVVTYALNTFTNLVVQLIRNMEAEEV<br>LEMQDLWLLRRSEKVTNWLQSNGWDRLKRMAVSGDDCVVKPIDDRFAHAL<br>RFLNDMGKVRKDTQEWKPSTGWDNWEEVPFCSHHFNKLHLKDGRSIVVPC<br>RHQDELIGRARVSPGAGWSIRETACLAKSYAQMWQLLYFHRRDLRLMANA<br>ICSSVPVDWVPTGRTTWSIHGKGEWMTTEDMLVVWNRVWIEENDHMEDKT<br>PVTKWTDIPYLGKREDLWCGSLIGHRPRTTWAENIKNTVNMVRRIIGDEE<br>KYMDYLSTQVRYLGEEGSTPGVL
ZIKV H/PF2013<br><br>Accession number:<br>KJ776791	MKNPKKKSGGFRIVNMLKRGVARVSPFGGLKRLPAGLLLGHGPIRMVLAI<br>LAFLRFTAIKPSLGLINRWGSVGKKEAME**I**IKKFKKDLAAMLRIINARKE<br>KKRRGADTSVGIVGLLLTTAMAAEVTRRGSAYYMYLDRNDAGEAISFPTT<br>LGMNKCYIQIMDLGHMCDATMSYECPMLDEGVEPDDVDCWCNTTSTWVVY<br>GTCHHKKGEARRSRRAVTLPSHSTRKLQTRSQTWLESREYTKHLIRVENW<br>IFRNPGFALAAAAIAWLLGSSTSQKVIYLVMILLIAPAYSIRCIGVSNRD<br>FVEGMSGGTWVDVVLEHGGCVTVMAQDKPTVDIELVTTTVSNMAEVRSYC<br>YEASISDMASDSRCPTQGEAYLDKQSDTQYVCKRTLVDRGWGNGCGLFGK<br>GSLVTCAKFACSKKMTGKSIQPENLEYRIMLSVHGSQHSGMIVNDTGHET<br>DENRAKVEITPNSPRAEATLGGFGSLGLDCEPRTGLDFSDLYYLTMNNKH<br>WLVHKEWFHDIPLPWHAGADTGTPHWNNKEALVEFKDAHAKRQTVVVLGS<br>QEGAVHTALAGALEAEMDGAKGRLSSGHLKCRLKMDKLRLKGVSYSLCTA<br>AFTFTKIPAETLHGTVTVEVQYAGTDGPCKVPAQMAVDMQTLTPVGRLIT<br>ANPVITESTENSKMMLELDPPFGDSYIVIGVGEKKITHHWHRSGSTIGKA<br>FEATVRGAKRMAVLGDTAWDFGSVGGALNSLGKGIHQIFGAAFKSLFGGM<br>SWFSQILIGTLLMWLGLNTKNGSISLMCLALGGVLIFLSTAVSADVGCSV<br>DFSKKETRCGTGVFVYNDVEAWRDRYKYHPDSPRRLAAAVKQAWEDGICG

	ISSVSRMENIMWRSVEGELNAILEENGVQLTVVVGSVKNPM**W**RGPQRLPV<br>PVNELPHGWKAWGKSYFVRAAKTNNSFVVDGDTLKECPLKHRAWNSFLVE<br>DHGFGVFHTSVWLKVREDYSLECDPAVIGTAVKGKEAVHSDLGYWIESEK<br>NDTWRLKRAHLIEMKTCEWPKSHTLWTDGIEESDLIIPKSLAGPLSHHNT<br>REGYRTQMKGPWHSEELEIRFEECPGTKVHVEETCGTRGPSLRSTTASGR<br>VIEEWCCRECTMPPLSFRAKDGCWYGMEIRPRKEPESNLVRSMVTAGSTD<br>HMDHFSLGVLVILLMVQEGLKKRMTTKIIISTSMAVLVAMILGGFSMSDL<br>AKLAILMGATFAEMNTGGDVAHLALIAAFKVRPALLVSFIFRANWTPRES<br>MLLALASCLLQTAISALEGDLMVLINGFALAWLAIRAMVVPRTDNITLAI<br>LAALTPLARGTLLVAWRAGLATCGGFMLLSLKGKGSVKKNLPFVMALGLT<br>AVRLVDPINVVGLLLLTRSGKRSWPPSEVLTAVGLICALAGGFAKADIEM<br>AGPMAAVGLLIVSYVVSGKSVDMYIERAGDITWEKDAEVTGNSPRLDVAL<br>DESGDFSLVEDDGPPMREIILKVVLMTICGMNPIAIPFAAGAWYVYVKTG<br>KRSGALWDVPAPKEVKKGETTDGVYRVMTRRLLGSTQVGVGVMQEGVFHT<br>MWHVTKGSALRSGEGRLDPYWGDVKQDLVSYCGPWKLDAAWDGHSEVQLL<br>AVPPGERARNIQTLPGIFKTKDGDIGAVALDYPAGTSGSPILDKCGRVIG<br>LYGNGVVIKNGSYVSAITQGRREEETPVECFEPSMLKKKQLTVLDLHPGA<br>GKTRRVLPEIVREAIKTRLRTVILAPTRVVAAEMEEALRGLPVRYMTTAV<br>NVTHSGTEIVDLMCHATFTSRLLQPIRVPNYNLYIMDEAHFTDPSSIAAR<br>GYISTRVEMGEAAAIFMTATPPGTRDAFPDSNSPIMDTEVEVPERAWSSG<br>FDWVTDHSGKTVWFVPSVRNGNEIAACLTKAGKRVIQLSRKTFETEFQKT<br>KHQEWDFVVTTDISEMGANFKADRVIDSRRCLKPVILDGERVILAGPMPV<br>THASAAQRRGRIGRNPNKPGDEYLYGGGCAETDEDHAHWLEARMLLDNIY<br>LQDGLIASLYRPEADKVAAIEGEFKLRTEQRKTFVELMKRGDLPVWLAYQ<br>VASAGITYTDRRWCFDGTTNNTIMEDSVPAEVWTRHGEKRVLKPRWMDAR<br>VCSDHAALKSFKEFAAGKRGAAFGVMEALGTLPGHMTERFQEAIDNLAVL<br>MRAETGSRPYKAAAAQLPETLETIMLLGLLGTVSLGIFFVLMRNKGIGKM<br>GFGMVTLGASAWLMWLSEIEPARIACVLIVVFLLLVVLIPEPEKQRSPQD<br>NQMAIIIMVAVGLLGLITANELGWLERTKSDLSHLMGRREEGATIGFSMD<br>IDLRPASAWAIYAALTTFITPAVQHAVTTSYNNYSLMAMATQAGVLFGMG<br>KGMPFYAWDFGVPLLMIGCYSQLTPLTLIVAIILLVAHYMYLIPGLQAAA<br>ARAAQKRTAAGIMKNPVVDGIVVTDIDTMTIDPQVEKKMGQVLLIAVAVS<br>SAILSRTAWGWGEAGALITAATSTLWEGSPNKYWNSSTATSLCNIFRGSY<br>LAGASLIYTVTRNAGLVKRRGGGTGETLGEKWKARLNQMSALEFYSYKKS<br>GITEVCREEARRALKDGVATGGHAVSRGSAKLRWLVERGYLQPYGKVIDL<br>GCGRGGWSYY**A**ATIRKVQEVKGYTKGGPGHEEP**M**LVQSYGWNIVRLKSGV<br>DVFHMAAEPCDTLLCDIGESSSSPEVEEARTLRVLSMVGDWLEKRPGAFC<br>IKVLCPYTSTMMETLERLQRRYGGGLVRVPLSRNSTHEMYWVSGAKSNTI<br>KSVSTTSQLLLGRMDGPRRPVKYEEDVNLGSGTRAVVSCAEAPNMKIIGN<br>RIERIRSEHAETWFFDENHPYRTWAYHGSYEAPTQGSASSLINGVVRLLS<br>KPWDVVTGVTGIAMTDTTPYGQQRVFKEKVDTRVPDPQEGTRQVMSMVSS<br>WLWKELGKHKRPRVCTKEEFINKVRSNAALGAIFEEEKEWKTAVEAVNDP<br>RFWALVDKEREHHLRGECQSCVYNMMGKREKKQGEFGKAKGSRAIWYMWL<br>GARFLEFEALGFLNEDHWMGRENSGGGVEGLGLQRLGYVLEEMSRIPGGR<br>MYADDTAGWDTRISRFDLENEALITNQMEKGHRALALAIIKYTYQNKVVK<br>VLRPAEKGKTVMDIISRQDQRGSGQVVTYALNTFTNLVVQLIRNMEAEEV<br>LEMQDLWLLRRSEKVTNWLQSNGWDRLKRMAVSGDDCVVKPIDDRFAHAL<br>RFLNDMGKVRKDTQEWKPSTGWDNWEEVPFCSHHFNKLHLKDGRSIVVPC<br>RHQDELIGRARVSPGAGWSIRETACLAKSYAQMWQLLYFHRRDLRLMANA<br>ICSSVPVDWVPTGRTTWSIHGKGEWMTTEDMLVVWNRVWIEENDHMEDKT<br>PVTKWTDIPYLGKREDLWCGSLIGHRPRTTWAENIKNTVNMVRRIIGDEE<br>KYMDYLSTQVRYLGEEGSTPGVL

Supplementary Table 2 | Antibody panel for Immunophenotyping.

Cell Subset	Ab	Clone	Dye	Company	Cat. #
B / T cells	CD20	2H7	PacificBlue	BioLegend	302328
	CD3	10D12	PE-Vio770	Miltenyi	130-104-202
	CD4	M-T466	PerCP	Miltenyi	130-101-147
	CD8	BW135/80	VioGreen	Miltenyi	130-096-902
	CD28	15E8	APC-Vio770	Miltenyi	130-104-278
	CD69	FN50	PE	BD	557050
	CD95	DX2	APC	Miltenyi	130-092-417
	Ki67	B56	Alexa 488	BD	558616
NK	CD3	10D12	APC	Miltenyi	130-091-998
	CD16	VEP13	APC-Vio770	Miltenyi	130-096-655
	CD56	AF12-7H3	PE	Miltenyi	130-090-755
	CD14	M5E2	V500	BD	561391
	CD8	SK1	BV421	BioLegend	344748
	NKp30	AF29-4D12	PE-Vio770	Miltenyi	130-104-116
	NKp46	BAB281	PC5	Beck-Coulter	A66902
	NK2GA	REA110	FITC	Miltenyi	130-098-818
DC	CD20	2H7	FITC	BD	555622
	CD3	SP34		BD	556611
	CD14	M5E2		BD	555397
	CD16	3G8		BD	555406
	NKG2A	REA110		Miltenyi	130-098-818
	CD8	SK1		BioLegend	344704
	HLA DR	REA 805	VioGreen	Miltenyi	130-111-795
	CD123	7G3	APC	BD	560087
	CD11c	3.9	PE/Cy7	BioLegend	301608

Supplementary Table 3 | Antibody panel for T cell functional response assessment.

Marker	Stain	Clone	Catalog Number	Vendor	Dilution
CD4	PerCP-Cy-5.5	SK3	566316	BD Biosciences	1:25
CD8β	PE	ECD	6607123	Beckman-Coulter	1:20
CD3	Pacific Blue	SP34-2	558124	BD Biosciences	1:30
CD20	BV605	2H7	563783	BD Biosciences	1:30
CD107a	FITC	H4A3	555800	BD Biosciences	1:10
CD28	PE-Cy-5	CD28.2	555730	BD Biosciences	1:10
CD95	BV510	DX2	305640	Biolegend	1:30
IFN-γ	APC	B27	554702	BD Biosciences	1:30
TNF-α	PE-Cy-7	Mab11	557647	BD Biosciences	1:30

Supplementary Table 4 | Peptide sequences for stimulation of T cell functional response.

Dengue Virus Type 2 Peptides

Peptide	Amino Acid Sequence	Peptide	Amino Acid Sequence	Peptide	Amino Acid Sequence
1	MRCIGISNRDFVEGV	29	AWLVHRQWFLDLPLPWL	57	MRGAKRMAILGDTAWDF
2	ISNRDFVEGVSGGSWVDI	30	WFLDLPLPWLPGADTQGSNW	58	AILGDTAWDFGSLGGVF
3	GVSGGSWVDIVLEHGSCV	31	PGADTQGSNWIQKETLV	59	WDFGSLGGVFTSIGKALH
4	DIVLEHGSCVTTMAKNK	32	SNWIQKETLVTFKNPHAK	60	VFTSIGKALHQVFGAIY
5	SCVTTMAKNKPTLDFELI	33	LVTFKNPHAKKQDVVVL	61	ALHQVFGAIYGAAFSGV
6	NKPTLDFELIETEAKQPA	34	HAKKQDVVVLGSQEGAMH	62	AIYGAAFSGVSWIMKILI
7	LIETEAKQPATLRKYCI	35	VLGSQEGAMHTALTGA	63	GVSWIMKILIGVIITWI
8	KQPATLRKYCIEAKL	36	GAMHTALTGATEIQM	64	ILIGVIITWIGMNSR
9	LRKYCIEAKLTNTTTDSR	37	ALTGATEIQMSSGNLLF	65	IITWIGMNSRSTSLSVSL
10	KLTNTTTDSRCPTQGEPSL	38	IQMSSGNLLFTGHLKCRL	66	SRSTSLSVSLVLVGVVTL
11	RCPTQGEPSLNEEQDKRF	39	LFTGHLKCRLRMDKLQLK	67	SLVLVGVVTLYLGVMVQA
12	SLNEEQDKRFVCKHSMV	40	RLRMDKLQLKGMSYSM		
13	KRFVCKHSMVDRGWGNGCG L	41	LQLKGMSYSMCTGKFKVV		
14	DRGWGNGCGLFGKGGIV	42	SMCTGKFKVVKEIAETQH		
15	CGLFGKGGIVTCAMFTCK	43	VVKEIAETQHGTIVIRV		
16	IVTCAMFTCKKNMKGKVV	44	TQHGTIVIRVQYEGDGSPCK		
17	CKKNMKGKVVQPENLEY	45	VQYEGDGSPCKIPFEIM		
18	KVVQPENLEYTIVITPH	46	SPCKIPFEIMDLEKRHVL		
19	LEYTIVITPHSGEEHAV	47	IMDLEKRHVLGRLITV		
20	TPHSGEEHAVGNDTGKH	48	RHVLGRLITVNPIVTEK		
21	HAVGNDTGKHGKEIKI	49	ITVNPIVTEKDSPVNIEA		
22	TGKHGKEIKITPQSSI	50	EKDSPVNIEAEPPFGDSY		
23	EIKITPQSSITEAELTGY	51	EAEPPFGDSYIIIGV		
24	SITEAELTGYGTVTM	52	FGDSYIIIGVEPGQLKL		
25	ELTGYGTVTMECSPRTGL	53	IGVEPGQLKLNWFKK		
26	TMECSPRTGLDFNEMVLL	54	GQLKLNWFKKGSSIGQMI		
27	GLDFNEMVLLQMENKAWL	55	KKGSSIGQMIETTMRGAK		
28	LLQMENKAWLVHRQWFL	56	MIETTMRGAKRMAIL		

Supplementary Table 4 | Continuation

Zika Virus Envelope Peptides

Peptide	Amino Acid Sequence	Peptide	Amino Acid Sequence	Peptide	Amino Acid Sequence
ZIKV59	IRCIGVSNRDFVEGM	ZIKV87	LSVHGSQHSGMIVND	ZIKV115	KGRLSSGHLKCRLKM
ZIKV60	VSNRDFVEGMSGGTW	ZIKV88	SQHSGMIVNDTGHET	ZIKV116	SGHLKCRLKMDKLRL
ZIKV61	FVEGMSGGTWVDVVL	ZIKV89	MIVNDTGHETDENRA	ZIKV117	CRLKMDKLRLKGVSY
ZIKV62	SGGTWVDVVLEHGGC	ZIKV90	TGHETDENRAKVEIT	ZIKV118	DKLRLKGVSYSLCTA
ZIKV63	VDVVLEHGGCVTVMA	ZIKV91	DENRAKVEITPNSPR	ZIKV119	KGVSYSLCTAAFTFT
ZIKV64	EHGGCVTVMAQDKPT	ZIKV92	KVEITPNSPRAEATL	ZIKV120	SLCTAAFTFTKIPAE
ZIKV65	VTVMAQDKPTVDIEL	ZIKV93	PNSPRAEATLGGFGS	ZIKV121	AFTFTKIPAETLHGT
ZIKV66	QDKPTVDIELVTTTV	ZIKV94	AEATLGGFGSLGLDC	ZIKV122	KIPAETLHGTVTVEV
ZIKV67	VDIELVTTTVSNMAE	ZIKV95	GGFGSLGLDCEPRTG	ZIKV123	TLHGTVTVEVQYAGT
ZIKV68	VTTTVSNMAEVRSYC	ZIKV96	LGLDCEPRTGLDFSD	ZIKV124	VTVEVQYAGTDGPCK
ZIKV69	SNMAEVRSYCYEASI	ZIKV97	EPRTGLDFSDLYYLT	ZIKV125	QYAGTDGPCKVPAQM
ZIKV70	VRSYCYEASISDMAS	ZIKV98	LDFSDLYYLTMNNKH	ZIKV126	DGPCKVPAQMAVDMQ
ZIKV71	YEASISDMASDSRCP	ZIKV99	LYYLTMNNKHWLVHK	ZIKV127	VPAQMAVDMQTLTPV
ZIKV72	SDMASDSRCPTQGEA	ZIKV100	MNNKHWLVHKEWFHD	ZIKV128	AVDMQTLTPVGRLIT
ZIKV73	DSRCPTQGEAYLDKQ	ZIKV101	WLVHKEWFHDIPLPW	ZIKV129	TLTPVGRLITANPVI
ZIKV74	TQGEAYLDKQSDTQY	ZIKV102	EWFHDIPLPWHAGAD	ZIKV130	GRLITANPVITESTE
ZIKV75	YLDKQSDTQYVCKRT	ZIKV103	IPLPWHAGADTGTPH	ZIKV131	ANPVITESTENSKMM
ZIKV76	SDTQYVCKRTLVDRG	ZIKV104	HAGADTGTPHWNNKE	ZIKV132	TESTENSKMMLELDP
ZIKV77	VCKRTLVDRGWGNGC	ZIKV105	TGTPHWNNKEALVEF	ZIKV133	NSKMMLELDPPFGDS
ZIKV78	LVDRGWGNGCGLFGK	ZIKV106	WNNKEALVEFKDAHA	ZIKV134	LELDPPFGDSYIVIG
ZIKV79	WGNGCGLFGKGSLVT	ZIKV107	ALVEFKDAHAKRQTV	ZIKV135	PFGDSYIVIGVGEKK
ZIKV80	GLFGKGSLVTCAKFA	ZIKV108	KDAHAKRQTVVVLGS	ZIKV136	YIVIGVGEKKITHHW
ZIKV81	GSLVTCAKFACSKKM	ZIKV109	KRQTVVVLGSQEGAV	ZIKV137	VGEKKITHHWHRSGS
ZIKV82	CAKFACSKKMTGKSI	ZIKV110	VVLGSQEGAVHTALA	ZIKV138	ITHHWHRSGSTIGKA
ZIKV83	CSKKMTGKSIQPENL	ZIKV111	QEGAVHTALAGALEA	ZIKV139	HRSGSTIGKAFEATV
ZIKV84	TGKSIQPENLEYRIM	ZIKV112	HTALAGALEAEMDGA	ZIKV140	TIGKAFEATVRGAKR
ZIKV85	QPENLEYRIMLSVHG	ZIKV113	GALEAEMDGAKGRLS	ZIKV141	FEATVRGAKRMAVLG
ZIKV86	EYRIMLSVHGSQHSG	ZIKV114	EMDGAKGRLSSGHLK	ZIKV142	RGAKRMAVLGDTAWD

Supplementary Table 4 | Continuation

Peptide	Amino Acid Sequence
ZIKV143	MAVLGDTAWDFGSVG
ZIKV144	DTAWDFGSVGGALNS
ZIKV145	FGSVGGALNSLGKGI
ZIKV146	GALNSLGKGIHQIFG
ZIKV147	LGKGIHQIFGAAFKS
ZIKV148	HQIFGAAFKSLFGGM
ZIKV149	AAFKSLFGGMSWFSQ
ZIKV150	LFGGMSWFSQILIGT
ZIKV151	SWFSQILIGTLLMWL
ZIKV152	ILIGTLLMWLGLNTK
ZIKV153	LLMWLGLNTKNGSIS
ZIKV154	GLNTKNGSISLMCLA
ZIKV155	NGSISLMCLALGGVL
ZIKV156	LMCLALGGVLIFLST
ZIKV157	LGGVLIFLSTAVSAD
ZIKV158	IFLSTAVSADVGCSV
ZIKV159	AVSADVGCSVDFSKK

Supplementary Table 4 | Continuation

Zika Virus Non-Structural Peptides

Peptide	Amino Acid Sequence	Peptide	Amino Acid Sequence	Peptide	Amino Acid Sequence
ZIKV160	VGCSVDFSKKETRCG	ZIKV188	ECPLKHRAWNSFLVE	ZIKV216	GTKVHVEETCGTRGP
ZIKV161	DFSKKETRCGTGVFV	ZIKV189	HRAWNSFLVEDHGFG	ZIKV217	VEETCGTRGPSLRST
ZIKV162	ETRCGTGVFVYNDVE	ZIKV190	SFLVEDHGFGVFHTS	ZIKV218	GTRGPSLRSTTASGR
ZIKV163	TGVFVYNDVEAWRDR	ZIKV191	DHGFGVFHTSVWLKV	ZIKV219	SLRSTTASGRVIEEW
ZIKV164	YNDVEAWRDRYKYHP	ZIKV192	VFHTSVWLKVREDYS	ZIKV220	TASGRVIEEWCCREC
ZIKV165	AWRDRYKYHPDSPRR	ZIKV193	VWLKVREDYSLECDP	ZIKV221	VIEEWCCRECTMPPL
ZIKV166	YKYHPDSPRRLAAAV	ZIKV194	REDYSLECDPAVIGT	ZIKV222	CCRECTMPPLSFRAK
ZIKV167	DSPRRLAAAVKQAWE	ZIKV195	LECDPAVIGTAVKGK	ZIKV223	TMPPLSFRAKDGCWY
ZIKV168	LAAAVKQAWEDGICG	ZIKV196	AVIGTAVKGKEAVHS	ZIKV224	SFRAKDGCWYGMEIR
ZIKV169	KQAWEDGICGISSVS	ZIKV197	AVKGKEAVHSDLGYW	ZIKV225	DGCWYGMEIRPRKEP
ZIKV170	DGICGISSVSRMENI	ZIKV198	EAVHSDLGYWIESEK	ZIKV226	GMEIRPRKEPESNLV
ZIKV171	ISSVSRMENIMWRSV	ZIKV199	DLGYWIESEKNDTWR	ZIKV227	PRKEPESNLVRSMVT
ZIKV172	RMENIMWRSVEGELN	ZIKV200	IESEKNDTWRLKRAH	ZIKV228	ESNLVRSMVTAGSTD
ZIKV173	MWRSVEGELNAILEE	ZIKV201	NDTWRLKRAHLIEMK	ZIKV229	RSMVTAGSTDHMDHF
ZIKV174	EGELNAILEENGVQL	ZIKV202	LKRAHLIEMKTCEWP	ZIKV230	AGSTDHMDHFSLGVL
ZIKV175	AILEENGVQLTVVVG	ZIKV203	LIEMKTCEWPKSHTL	ZIKV231	HMDHFSLGVLVILLM
ZIKV176	NGVQLTVVVGSVKNP	ZIKV204	TCEWPKSHTLWTDGI	ZIKV232	SLGVLVILLMVQEGL
ZIKV177	TVVVGSVKNPMWRGP	ZIKV205	KSHTLWTDGIEESDL	ZIKV233	VILLMVQEGLKKRMT
ZIKV178	SVKNPMWRGPQRLPV	ZIKV206	WTDGIEESDLIIPKS	ZIKV234	VQEGLKKRMTTKIII
ZIKV179	MWRGPQRLPVPVNEL	ZIKV207	EESDLIIPKSLAGPL	ZIKV235	KKRMTTKIIISTSMA
ZIKV180	QRLPVPVNELPHGWK	ZIKV208	IIPKSLAGPLSHHNT	ZIKV236	TKIIISTSMAVLVAM
ZIKV181	PVNELPHGWKAWGKS	ZIKV209	LAGPLSHHNTREGYR	ZIKV237	STSMAVLVAMILGGF
ZIKV182	PHGWKAWGKSYFVRA	ZIKV210	SHHNTREGYRTQMKG	ZIKV238	VLVAMILGGFSMSDL
ZIKV183	AWGKSYFVRAAKTNN	ZIKV211	REGYRTQMKGPWHSE	ZIKV239	ILGGFSMSDLAKLAI
ZIKV184	YFVRAAKTNNSFVVD	ZIKV212	TQMKGPWHSEELEIR	ZIKV240	SMSDLAKLAILMGAT
ZIKV185	AKTNNSFVVDGDTLK	ZIKV213	PWHSEELEIRFEECP	ZIKV241	AKLAILMGATFAEMN
ZIKV186	SFVVDGDTLKECPLK	ZIKV214	ELEIRFEECPGTKVH	ZIKV242	LMGATFAEMNTGGDV
ZIKV187	GDTLKECPLKHRAWN	ZIKV215	FEECPGTKVHVEETC	ZIKV243	FAEMNTGGDVAHLAL

Peptide	Amino Acid Sequence	Peptide	Amino Acid Sequence	Peptide	Amino Acid Sequence
ZIKV244	TGGDVAHLALIAAFK	ZIKV272	DPINVVGLLLLTRSG	ZIKV300	YVKTGKRSGALWDVP
ZIKV245	AHLALIAAFKVRPAL	ZIKV273	VGLLLLTRSGKRSWP	ZIKV301	KRSGALWDVPAPKEV
ZIKV246	IAAFKVRPALLVSFI	ZIKV274	LTRSGKRSWPPSEVL	ZIKV302	LWDVPAPKEVKKGET
ZIKV247	VRPALLVSFIFRANW	ZIKV275	KRSWPPSEVLTAVGL	ZIKV303	APKEVKKGETTDGVY
ZIKV248	LVSFIFRANWTPRES	ZIKV276	PSEVLTAVGLICALA	ZIKV304	KKGETTDGVYRVMTR
ZIKV249	FRANWTPRESMLLAL	ZIKV277	TAVGLICALAGGFAK	ZIKV305	TDGVYRVMTRRLLGS
ZIKV250	TPRESMLLALASCLL	ZIKV278	ICALAGGFAKADIEM	ZIKV306	RVMTRRLLGSTQVGV
ZIKV251	MLLALASCLLQTAIS	ZIKV279	GGFAKADIEMAGPMA	ZIKV307	RLLGSTQVGVGVMQE
ZIKV252	ASCLLQTAISALEGD	ZIKV280	ADIEMAGPMAAVGLL	ZIKV308	TQVGVGVMQEGVFHT
ZIKV253	QTAISALEGDLMVLI	ZIKV281	AGPMAAVGLLIVSYV	ZIKV309	GVMQEGVFHTMWHVT
ZIKV254	ALEGDLMVLINGFAL	ZIKV282	AVGLLIVSYVVSGKS	ZIKV310	GVFHTMWHVTKGSAL
ZIKV255	LMVLINGFALAWLAI	ZIKV283	IVSYVVSGKSVDMYI	ZIKV311	MWHVTKGSALRSGEG
ZIKV256	NGFALAWLAIRAMVV	ZIKV284	VSGKSVDMYIERAGD	ZIKV312	KGSALRSGEGRLDPY
ZIKV257	AWLAIRAMVVPRTDN	ZIKV285	VDMYIERAGDITWEK	ZIKV313	RSGEGRLDPYWGDVK
ZIKV258	RAMVVPRTDNITLAI	ZIKV286	ERAGDITWEKDAEVT	ZIKV314	RLDPYWGDVKQDLVS
ZIKV259	PRTDNITLAILAALT	ZIKV287	ITWEKDAEVTGNSPR	ZIKV315	WGDVKQDLVSYCGPW
ZIKV260	ITLAILAALTPLARG	ZIKV288	DAEVTGNSPRLDVAL	ZIKV316	QDLVSYCGPWKLDAA
ZIKV261	LAALTPLARGTLLVA	ZIKV289	GNSPRLDVALDESGD	ZIKV317	YCGPWKLDAAWDGHS
ZIKV262	PLARGTLLVAWRAGL	ZIKV290	LDVALDESGDFSLVE	ZIKV318	KLDAAWDGHSEVQLL
ZIKV263	TLLVAWRAGLATCGG	ZIKV291	DESGDFSLVEDDGPP	ZIKV319	WDGHSEVQLLAVPPG
ZIKV264	WRAGLATCGGFMLLS	ZIKV292	FSLVEDDGPPMREII	ZIKV320	EVQLLAVPPGERARN
ZIKV265	ATCGGFMLLSLKGKG	ZIKV293	DDGPPMREIILKVVL	ZIKV321	AVPPGERARNIQTLP
ZIKV266	FMLLSLKGKGSVKKN	ZIKV294	MREIILKVVLMTICG	ZIKV322	ERARNIQTLPGIFKT
ZIKV267	LKGKGSVKKNLPFVM	ZIKV295	LKVVLMTICGMNPIA	ZIKV323	IQTLPGIFKTKDGDI
ZIKV268	SVKKNLPFVMALGLT	ZIKV296	MTICGMNPIAIPFAA	ZIKV324	GIFKTKDGDIGAVAL
ZIKV269	LPFVMALGLTAVRLV	ZIKV297	MNPIAIPFAAGAWYV	ZIKV325	KDGDIGAVALDYPAG
ZIKV270	ALGLTAVRLVDPINV	ZIKV298	IPFAAGAWYVYVKTG	ZIKV326	GAVALDYPAGTSGSP
ZIKV271	AVRLVDPINVVGLLL	ZIKV299	GAWYVYVKTGKRSGA	ZIKV327	DYPAGTSGSPILDKC

Peptide	Amino Acid Sequence	Peptide	Amino Acid Sequence	Peptide	Amino Acid Sequence
ZIKV328	TSGSPILDKCGRVIG	ZIKV356	IRVPNYNLYIMDEAH	ZIKV384	MGANFKADRVIDSRR
ZIKV329	ILDKCGRVIGLYGNG	ZIKV357	YNLYIMDEAHFTDPS	ZIKV385	KADRVIDSRRCLKPV
ZIKV330	GRVIGLYGNGVVIKN	ZIKV358	MDEAHFTDPSSIAAR	ZIKV386	IDSRRCLKPVILDGE
ZIKV331	LYGNGVVIKNGSYVS	ZIKV359	FTDPSSIAARGYIST	ZIKV387	CLKPVILDGERVILA
ZIKV332	VVIKNGSYVSAITQG	ZIKV360	SIAARGYISTRVEMG	ZIKV388	ILDGERVILAGPMPV
ZIKV333	GSYVSAITQGRREEE	ZIKV361	GYISTRVEMGEAAAI	ZIKV389	RVILAGPMPVTHASA
ZIKV334	AITQGRREEETPVEC	ZIKV362	RVEMGEAAAIFMTAT	ZIKV390	GPMPVTHASAAQRRG
ZIKV335	RREEETPVECFEPSM	ZIKV363	EAAAIFMTATPPGTR	ZIKV391	THASAAQRRGRIGRN
ZIKV336	TPVECFEPSMLKKKQ	ZIKV364	FMTATPPGTRDAFPD	ZIKV392	AQRRGRIGRNPNKPG
ZIKV337	FEPSMLKKKQLTVLD	ZIKV365	PPGTRDAFPDSNSPI	ZIKV393	RIGRNPNKPGDEYLY
ZIKV338	LKKKQLTVLDLHPGA	ZIKV366	DAFPDSNSPIMDTEV	ZIKV394	PNKPGDEYLYGGGCA
ZIKV339	LTVLDLHPGAGKTRR	ZIKV367	SNSPIMDTEVEVPER	ZIKV395	DEYLYGGGCAETDED
ZIKV340	LHPGAGKTRRVLPEI	ZIKV368	MDTEVEVPERAWSSG	ZIKV396	GGGCAETDEDHAHWL
ZIKV341	GKTRRVLPEIVREAI	ZIKV369	EVPERAWSSGFDWVT	ZIKV397	ETDEDHAHWLEARML
ZIKV342	VLPEIVREAIKTRLR	ZIKV370	AWSSGFDWVTDHSGK	ZIKV398	HAHWLEARMLLDNIY
ZIKV343	VREAIKTRLRTVILA	ZIKV371	FDWVTDHSGKTVWFV	ZIKV399	EARMLLDNIYLQDGL
ZIKV344	KTRLRTVILAPTRVV	ZIKV372	DHSGKTVWFVPSVRN	ZIKV400	LDNIYLQDGLIASLY
ZIKV345	TVILAPTRVVAAEME	ZIKV373	TVWFVPSVRNGNEIA	ZIKV401	LQDGLIASLYRPEAD
ZIKV346	PTRVVAAEMEEALRG	ZIKV374	PSVRNGNEIAACLTK	ZIKV402	IASLYRPEADKVAAI
ZIKV347	AAEMEEALRGLPVRY	ZIKV375	GNEIAACLTKAGKRV	ZIKV403	RPEADKVAAIEGEFK
ZIKV348	EALRGLPVRYMTTAV	ZIKV376	ACLTKAGKRVIQLSR	ZIKV404	KVAAIEGEFKLRTEQ
ZIKV349	LPVRYMTTAVNVTHS	ZIKV377	AGKRVIQLSRKTFET	ZIKV405	EGEFKLRTEQRKTFV
ZIKV350	MTTAVNVTHSGTEIV	ZIKV378	IQLSRKTFETEFQKT	ZIKV406	LRTEQRKTFVELMKR
ZIKV351	NVTHSGTEIVDLMCH	ZIKV379	KTFETEFQKTKHQEW	ZIKV407	RKTFVELMKRGDLPV
ZIKV352	GTEIVDLMCHATFTS	ZIKV380	EFQKTKHQEWDFVVT	ZIKV408	ELMKRGDLPVWLAYQ
ZIKV353	DLMCHATFTSRLLQP	ZIKV381	KHQEWDFVVTTDISE	ZIKV409	GDLPVWLAYQVASAG
ZIKV354	ATFTSRLLQPIRVPN	ZIKV382	DFVVTTDISEMGANF	ZIKV410	WLAYQVASAGITYTD
ZIKV355	RLLQPIRVPNYNLYI	ZIKV383	TDISEMGANFKADRV	ZIKV411	VASAGITYTDRRWCF

Peptide	Amino Acid Sequence	Peptide	Amino Acid Sequence	Peptide	Amino Acid Sequence
ZIKV412	ITYTDRRWCFDGTTN	ZIKV440	GIGKMGFGMVTLGAS	ZIKV468	LMAMATQAGVLFGMG
ZIKV413	RRWCFDGTTNNTIME	ZIKV441	GFGMVTLGASAWLMW	ZIKV469	TQAGVLFGMGKGMPF
ZIKV414	DGTTNNTIMEDSVPA	ZIKV442	TLGASAWLMWLSEIE	ZIKV470	LFGMGKGMPFYAWDF
ZIKV415	NTIMEDSVPAEVWTR	ZIKV443	AWLMWLSEIEPARIA	ZIKV471	KGMPFYAWDFGVPLL
ZIKV416	DSVPAEVWTRHGEKR	ZIKV444	LSEIEPARIACVLIV	ZIKV472	YAWDFGVPLLMIGCY
ZIKV417	EVWTRHGEKRVLKPR	ZIKV445	PARIACVLIVVFLLL	ZIKV473	GVPLLMIGCYSQLTP
ZIKV418	HGEKRVLKPRWMDAR	ZIKV446	CVLIVVFLLLVVLIP	ZIKV474	MIGCYSQLTPLTLIV
ZIKV419	VLKPRWMDARVCSDH	ZIKV447	VFLLLVVLIPEPEKQ	ZIKV475	SQLTPLTLIVAIILL
ZIKV420	WMDARVCSDHAALKS	ZIKV448	VVLIPEPEKQRSPQD	ZIKV476	LTLIVAIILLVAHYM
ZIKV421	VCSDHAALKSFKEFA	ZIKV449	EPEKQRSPQDNQMAI	ZIKV477	AIILLVAHYMYLIPG
ZIKV422	AALKSFKEFAAGKRG	ZIKV450	RSPQDNQMAIIIMVA	ZIKV478	VAHYMYLIPGLQAAA
ZIKV423	FKEFAAGKRGAAFGV	ZIKV451	NQMAIIIMVAVGLLG	ZIKV479	YLIPGLQAAAARAAQ
ZIKV424	AGKRGAAFGVMEALG	ZIKV452	IIMVAVGLLGLITAN	ZIKV480	LQAAAARAAQKRTAA
ZIKV425	AAFGVMEALGTLPGH	ZIKV453	VGLLGLITANELGWL	ZIKV481	ARAAQKRTAAGIMKN
ZIKV426	MEALGTLPGHMTERF	ZIKV454	LITANELGWLERTKS	ZIKV482	KRTAAGIMKNPVVDG
ZIKV427	TLPGHMTERFQEAID	ZIKV455	ELGWLERTKSDLSHL	ZIKV483	GIMKNPVVDGIVVTD
ZIKV428	MTERFQEAIDNLAVL	ZIKV456	ERTKSDLSHLMGRRE	ZIKV484	PVVDGIVVTDIDTMT
ZIKV429	QEAIDNLAVLMRAET	ZIKV457	DLSHLMGRREEGATI	ZIKV485	IVVTDIDTMTIDPQV
ZIKV430	NLAVLMRAETGSRPY	ZIKV458	MGRREEGATIGFSMD	ZIKV486	IDTMTIDPQVEKKMG
ZIKV431	MRAETGSRPYKAAAA	ZIKV459	EGATIGFSMDIDLRP	ZIKV487	IDPQVEKKMGQVLLI
ZIKV432	GSRPYKAAAAQLPET	ZIKV460	GFSMDIDLRPASAWA	ZIKV488	EKKMGQVLLIAVAVS
ZIKV433	KAAAAQLPETLETIM	ZIKV461	IDLRPASAWAIYAAL	ZIKV489	QVLLIAVAVSSAILS
ZIKV434	QLPETLETIMLLGLL	ZIKV462	ASAWAIYAALTTFIT	ZIKV490	AVAVSSAILSRTAWG
ZIKV435	LETIMLLGLLGTVSL	ZIKV463	IYAALTTFITPAVQH	ZIKV491	SAILSRTAWGWGEAG
ZIKV436	LLGLLGTVSLGIFFV	ZIKV464	TTFITPAVQHAVTTS	ZIKV492	RTAWGWGEAGALITA
ZIKV437	GTVSLGIFFVLMRNK	ZIKV465	PAVQHAVTTSYNNYS	ZIKV493	WGEAGALITAATSTL
ZIKV438	GIFFVLMRNKGIGKM	ZIKV466	AVTTSYNNYSLMAMA	ZIKV494	ALITAATSTLWEGSP
ZIKV439	LMRNKGIGKMGFGMV	ZIKV467	YNNYSLMAMATQAGV	ZIKV495	ATSTLWEGSPNKYWN

Peptide	Amino Acid Sequence	Peptide	Amino Acid Sequence	Peptide	Amino Acid Sequence
ZIKV496	WEGSPNKYWNSSTAT	ZIKV524	KVQEVKGYTKGGPGH	ZIKV552	TSQLLLGRMDGPRRP
ZIKV497	NKYWNSSTATSLCNI	ZIKV525	KGYTKGGPGHEEPVL	ZIKV553	LGRMDGPRRPVKYEE
ZIKV498	SSTATSLCNIFRGSY	ZIKV526	GGPGHEEPVLVQSYG	ZIKV554	GPRRPVKYEEDVNLG
ZIKV499	SLCNIFRGSYLAGAS	ZIKV527	EEPVLVQSYGWNIVR	ZIKV555	VKYEEDVNLGSGTRA
ZIKV500	FRGSYLAGASLIYTV	ZIKV528	VQSYGWNIVRLKSGV	ZIKV556	DVNLGSGTRAVVSCA
ZIKV501	LAGASLIYTVTRNAG	ZIKV529	WNIVRLKSGVDVFHM	ZIKV557	SGTRAVVSCAEAPNM
ZIKV502	LIYTVTRNAGLVKRR	ZIKV530	LKSGVDVFHMAAEPC	ZIKV558	VVSCAEAPNMKIIGN
ZIKV503	TRNAGLVKRRGGGTG	ZIKV531	DVFHMAAEPCDTLLC	ZIKV559	EAPNMKIIGNRIERI
ZIKV504	LVKRRGGGTGETLGE	ZIKV532	AAEPCDTLLCDIGES	ZIKV560	KIIGNRIERIRSEHA
ZIKV505	GGGTGETLGEKWKAR	ZIKV533	DTLLCDIGESSSSPE	ZIKV561	RIERIRSEHAETWFF
ZIKV506	ETLGEKWKARLNQMS	ZIKV534	DIGESSSSPEVEEAR	ZIKV562	RSEHAETWFFDENHP
ZIKV507	KWKARLNQMSALEFY	ZIKV535	SSSPEVEEARTLRVL	ZIKV563	ETWFFDENHPYRTWA
ZIKV508	LNQMSALEFYSYKKS	ZIKV536	VEEARTLRVLSMVGD	ZIKV564	DENHPYRTWAYHGSY
ZIKV509	ALEFYSYKKSGITEV	ZIKV537	TLRVLSMVGDWLEKR	ZIKV565	YRTWAYHGSYEAPTQ
ZIKV510	SYKKSGITEVCREEA	ZIKV538	SMVGDWLEKRPGAFC	ZIKV566	YHGSYEAPTQGSASS
ZIKV511	GITEVCREEARRALK	ZIKV539	WLEKRPGAFCIKVLC	ZIKV567	EAPTQGSASSLINGV
ZIKV512	CREEARRALKDGVAT	ZIKV540	PGAFCIKVLCPYTST	ZIKV568	GSASSLINGVVRLLS
ZIKV513	RRALKDGVATGGHAV	ZIKV541	IKVLCPYTSTMMETL	ZIKV569	LINGVVRLLSKPWDV
ZIKV514	DGVATGGHAVSRGSA	ZIKV542	PYTSTMMETLERLQR	ZIKV570	VRLLSKPWDVVTGVT
ZIKV515	GGHAVSRGSAKLRWL	ZIKV543	MMETLERLQRRYGGG	ZIKV571	KPWDVVTGVTGIAMT
ZIKV516	SRGSAKLRWLVERGY	ZIKV544	ERLQRRYGGGLVRVP	ZIKV572	VTGVTGIAMTDTTPY
ZIKV517	KLRWLVERGYLQPYG	ZIKV545	RYGGGLVRVPLSRNS	ZIKV573	GIAMTDTTPYGQQRV
ZIKV518	VERGYLQPYGKVIDL	ZIKV546	LVRVPLSRNSTHEMY	ZIKV574	DTTPYGQQRVFKEKV
ZIKV519	LQPYGKVIDLGCGRG	ZIKV547	LSRNSTHEMYWVSGA	ZIKV575	GQQRVFKEKVDTRVP
ZIKV520	KVIDLGCGRGGWSYY	ZIKV548	THEMYWVSGAKSNTI	ZIKV576	FKEKVDTRVPDPQEG
ZIKV521	GCGRGGWSYYVATIR	ZIKV549	WVSGAKSNTIKSVST	ZIKV577	DTRVPDPQEGTRQVM
ZIKV522	GWSYYVATIRKVQEV	ZIKV550	KSNTIKSVSTTSQLL	ZIKV578	DPQEGTRQVMSMVSS
ZIKV523	VATIRKVQEVKGYTK	ZIKV551	KSVSTTSQLLLGRMD	ZIKV579	TRQVMSMVSSWLWKE

Peptide	Amino Acid Sequence	Peptide	Amino Acid Sequence	Peptide	Amino Acid Sequence
ZIKV580	SMVSSWLWKELGKHK	ZIKV608	LGYVLEEMSRIPGGR	ZIKV636	RLKRMAVSGDDCVVK
ZIKV581	WLWKELGKHKRPRVC	ZIKV609	EEMSRIPGGRMYADD	ZIKV637	AVSGDDCVVKPIDDR
ZIKV582	LGKHKRPRVCTKEEF	ZIKV610	IPGGRMYADDTAGWD	ZIKV638	DCVVKPIDDRFAHAL
ZIKV583	RPRVCTKEEFINKVR	ZIKV611	MYADDTAGWDTRISR	ZIKV639	PIDDRFAHALRFLND
ZIKV584	TKEEFINKVRSNAAL	ZIKV612	TAGWDTRISRFDLEN	ZIKV640	FAHALRFLNDMGKVR
ZIKV585	INKVRSNAALGAIFE	ZIKV613	TRISRFDLENEALIT	ZIKV641	RFLNDMGKVRKDTQE
ZIKV586	SNAALGAIFEEEKEW	ZIKV614	FDLENEALITNQMEK	ZIKV642	MGKVRKDTQEWKPST
ZIKV587	GAIFEEEKEWKTAVE	ZIKV615	EALITNQMEKGHRAL	ZIKV643	KDTQEWKPSTGWDNW
ZIKV588	EEKEWKTAVEAVNDP	ZIKV616	NQMEKGHRALALAII	ZIKV644	WKPSTGWDNWEEVPF
ZIKV589	KTAVEAVNDPRFWAL	ZIKV617	GHRALALAIIKYTYQ	ZIKV645	GWDNWEEVPFCSHHF
ZIKV590	AVNDPRFWALVDKER	ZIKV618	ALAIIKYTYQNKVVK	ZIKV646	EEVPFCSHHFNKLHL
ZIKV591	RFWALVDKEREHHLR	ZIKV619	KYTYQNKVVKVLRPA	ZIKV647	CSHHFNKLHLKDGRS
ZIKV592	VDKEREHHLRGECQS	ZIKV620	NKVVKVLRPAEKGKT	ZIKV648	NKLHLKDGRSIVVPC
ZIKV593	EHHLRGECQSCVYNM	ZIKV621	VLRPAEKGKTVMDII	ZIKV649	KDGRSIVVPCRHQDE
ZIKV594	GECQSCVYNMMGKRE	ZIKV622	EKGKTVMDIISRQDQ	ZIKV650	IVVPCRHQDELIGRA
ZIKV595	CVYNMMGKREKKQGE	ZIKV623	VMDIISRQDQRGSGQ	ZIKV651	RHQDELIGRARVSPG
ZIKV596	MGKREKKQGEFGKAK	ZIKV624	SRQDQRGSGQVVTYA	ZIKV652	LIGRARVSPGAGWSI
ZIKV597	KKQGEFGKAKGSRAI	ZIKV625	RGSGQVVTYALNTFT	ZIKV653	RVSPGAGWSIRETAC
ZIKV598	FGKAKGSRAIWYMWL	ZIKV626	VVTYALNTFTNLVVQ	ZIKV654	AGWSIRETACLAKSY
ZIKV599	GSRAIWYMWLGARFL	ZIKV627	LNTFTNLVVQLIRNM	ZIKV655	RETACLAKSYAQMWQ
ZIKV600	WYMWLGARFLEFEAL	ZIKV628	NLVVQLIRNMEAEEV	ZIKV656	LAKSYAQMWQLLYFH
ZIKV601	GARFLEFEALGFLNE	ZIKV629	LIRNMEAEEVLEMQD	ZIKV657	AQMWQLLYFHRRDLR
ZIKV602	EFEALGFLNEDHWMG	ZIKV630	EAEEVLEMQDLWLLR	ZIKV658	LLYFHRRDLRLMANA
ZIKV603	GFLNEDHWMGRENSG	ZIKV631	LEMQDLWLLRRSEKV	ZIKV659	RRDLRLMANAICSSV
ZIKV604	DHWMGRENSGGGVEG	ZIKV632	LWLLRRSEKVTNWLQ	ZIKV660	LMANAICSSVPVDWV
ZIKV605	RENSGGGVEGLGLQR	ZIKV633	RSEKVTNWLQSNGWD	ZIKV661	ICSSVPVDWVPTGRT
ZIKV606	GGVEGLGLQRLGYVL	ZIKV634	TNWLQSNGWDRLKRM	ZIKV662	PVDWVPTGRTTWSIH
ZIKV607	LGLQRLGYVLEEMSR	ZIKV635	SNGWDRLKRMAVSGD	ZIKV663	PTGRTTWSIHGKGEW

Peptide	Amino Acid Sequence
ZIKV664	TWSIHGKGEWMTTED
ZIKV665	GKGEWMTTEDMLVVW
ZIKV666	MTTEDMLVVWNRVWI
ZIKV667	MLVVWNRVWIEENDH
ZIKV668	NRVWIEENDHMEDKT
ZIKV669	EENDHMEDKTPVTKW
ZIKV670	MEDKTPVTKWTDIPY
ZIKV671	PVTKWTDIPYLGKRE
ZIKV672	TDIPYLGKREDLWCG
ZIKV673	LGKREDLWCGSLIGH
ZIKV674	DLWCGSLIGHRPRTT
ZIKV675	SLIGHRPRTTWAENI
ZIKV676	RPRTTWAENIKNTVN
ZIKV677	WAENIKNTVNMVRRI
ZIKV678	KNTVNMVRRIIGDEE
ZIKV679	MVRRIIGDEEKYMDY
ZIKV680	IGDEEKYMDYLSTQV
ZIKV681	KYMDYLSTQVRYLGE
ZIKV682	LSTQVRYLGEEGSTP
ZIKV683	RYLGEEGSTPGVL

Supplementary Discussion

Findings summary

In summary, dissecting our main findings per previous ZIKV-immune status we found that a ZIKV middle-convalescence: (i) results in shorter DENV viremic period, (ii) lowest pro-inflammatory status with upregulation of cellular immune response mediators, (iii) robust neutralizing antibody response higher in magnitude and durability against ZIKV strains and DENV serotypes, (iv) elevated activated and proliferating B cells, (v) early activation of cross-reactive $CD4^+$ and $CD8^+$ effector memory T cells, (v) and a major breadth of functional T cell response. For ZIKV early-convalescence we demonstrated: (i) average DENV viremic period and no exacerbation of pro-inflammatory status, (ii) neutralizing antibody response with high magnitude but less durability against ZIKV strains and DENV serotypes compared to the ZIKV middle-convalescent group, (iii) early activation of central memory $CD8^+$ T cells, (iv) and very limited activation of effector memory T cells. For the ZIKV-naïve group we demonstrated: (i) longer DENV viremic period and pro-inflammatory status, (ii) a more delayed *de novo* neutralizing antibody response against DENV serotypes and inability to neutralize ZIKV strains, (iii) a limited B cell response, (iv) and an overall *de novo* T cell response lower in magnitude and cross-reactivity compared to ZIKV-immune groups.

DENV RNAemia

The lack of significant DENV RNAemia enhancement and pro-inflammatory status in ZIKV immune animals in our work, compared to *George et al.*, may be attributable to the different sample types collected (plasma vs serum), or different DENV-2 strains used for the challenge (New Guinea/1944 strain vs Thailand/16681/1964 strain, from Asian II and Asian I Genotype, respectively). The strains fact is of relevance because it suggests that the effect of previous ZIKV immunity on a subsequent DENV infection may differ between DENV serotypes or even within genotypes. Another possible explanation is the genetic heterogeneity of rhesus macaques used in these two studies as they are derived from different breeders. The importance of selecting genetic well-characterized macaques have been discussed previously[1].

Cytokine profile

A lack of ZIKV immunity promoted a more pro-inflammatory profile after DENV infection characterized by significant elevated levels of IL-6 and MIG/CXCL9. Interestingly, higher levels of IFN-α were observed in the ZIKV-naïve animals. This antiviral cytokine is known to be actively produced during acute DENV infection *in vitro* and *in vivo*[2]. Elevated levels have been correlated with severity in DHF patients, and to act as a marker for elevated DENV replication[3,4]. On the other hand, the presence of a longer ZIKV convalescence is associated with increased levels of CXCL10 and perforin. CXCL10 is an immune mediator for T cells proliferation, recruitment of $CD4^+$ and $CD8^+$ activated T cells and IFN-γ-producing $CD8^+$ T cells, required to control DENV infection *in vivo*[5,6]. This correlates with higher proportion and activation of both T cell compartments and subsequent functional T cell response against DENV-E-specific peptides

in the group with longer convalescence to ZIKV. Perforin is involved in the cytotoxic degranulation process against virus-infected cells. In DENV infection, perforin is part of the anti-DENV cytotoxic phenotype of $CD8^+$ and $CD4^+$ T cells[7,8]. Perforin levels were significantly elevated only in the ZIKV mid-convalescent group after DENV infection. Accordingly, this coincides with a significant activation of $CD8^+$ and $CD4^+$ effector memory T cells, and degranulation functional response of both T cell compartments, suggesting an enhanced perforin-producing cytotoxic role of T cells in presence of longer convalescence to ZIKV. Contrary to our findings, a previously published work found that an approximately two month ZIKV immunity period resulted in an increase of pro-inflammatory cytokines[9]. However, a differential effect due to the use of different sample types (plasma vs serum) between both studies cannot be ruled out.

Neutralizing antibody response

Is still uncertain why the ZIKVPF-10mo animals have a slightly higher peak of Ab response compared to the ZIKVPR-2mo animals. We speculate this may be caused by modification of MBCs overtime, so that by 10 months the cells are able to better respond to antigen compared to cells at two months. After ZIKV infection in human DENV-naïve subjects, the ZIKV/DENV cross-reactive MBC response increased in magnitude (39% of total MBC proportion) after longer periods of ZIKV convalescence (~8 months post-ZIKV infection)[10], similar to the 10 months in the ZIKV mid-convalescent group that exhibited higher DENV cross-neutralization. Based upon studies of human monoclonal Abs, plasmablasts response during secondary DENV infection is mainly of MBC origin, resulting in a mature response characterized by cross-neutralizing Abs *in vitro*[11]. These are seminal contributions to forecast and understand the cross-neutralization capacity of further heterologous DENV epidemics in the context of previous ZIKV-DENV immunity. Interestingly, ZIKV-convalescent animals showed some degree of cross-neutralization against DENV-2 and DENV-4 before DENV infection. This is consistent with our previous results showing that DENV-naïve ZIKV-infected animals also preferentially neutralized DENV-4 followed by DENV-2 after ZIKV infection[12]. Longitudinal data of cross-neutralization of DENV serotypes in DENV-naïve ZIKV-infected human subjects showed low cross-neutralization against all DENV serotypes, but DENV-4 followed by DENV-2 were neutralized more efficiently up to 6 months after ZIKV infection with comparable basal titers reported here[13]. There is no data yet that delineates shared cross-neutralizing epitopes between ZIKV and DENV-2/-4, but it is known that DENV-4 genotypic diversity impact the capacity of its neutralization[14]. On the other hand, we showed that naïve animals with DENV *de novo* response did not cross-neutralized ZIKV at all, which state that although similar, antigenic differences are sufficient to mount predominantly type-specific rather than cross-reactive responses during a primary infection[10,15].

T cells phenotyping

The ZIKV early-convalescent group displays a modest activation (T-CM>T-EM) early after DENV infection (Supplementary Discussion: T cells phenotyping). Since this group was infected with ZIKV only two months before DENV it is possible that after viral clearance and

development of ZIKV-specific T cell response, the T cell compartments were still under the contraction phase at the time of the DENV challenge. Yellow fever virus (YFV) and vaccinia virus vaccinations in humans demonstrate that T cell contraction start as early as approximately one-month post-vaccination and at least for almost three months is still ongoing[16]. Also, a study shows that re-stimulation using alphavirus replicons during T cell response contraction does not have significant impact modulating the pre-existing T cell response[17].

T cells functional response

Strikingly, this response recognizes more efficiently peptides from DENV E protein than from ZIKV E protein. ZIKV-specific $CD8^+$ T cells direct 57% of their response against structural proteins, which may suggest these cells can recognize conserved epitopes between ZIKV and DENV structural proteins. Cross-reactivity of T cells between heterologous flavivirus infections is explained by selective immune recall of memory T cells that recognize conserved epitopes between DENV and ZIKV[18], which also has previously been demonstrated during secondary heterotypic DENV infections[19,20].

Higher proportion of IFN-γ and TNF-α producing T cells before a secondary heterologous DENV infection has been associated to a subsequent subclinical outcome[21]. Herein, we observed that the ZIKV mid-convalescent group had elevated levels of IFN-γ and TNF-α producing T cells since baseline. In this group, DENV infection stimulated a higher frequency of these cells, but remarkably, also increased highly cross-reactive IFN-γ-producing $CD4^+$ T cells directed to DENV E, and ZIKV E/NS proteins.

Supplementary References

1 Kanthaswamy, S. *et al.* The Population Genetic Composition of Conventional and SPF Colonies of Rhesus Macaques (Macaca mulatta) at the Caribbean Primate Research Center. *Journal of the American Association for Laboratory Animal Science : JAALAS* **55**, 147-151 (2016).

2 Gandini, M. *et al.* Dengue virus activates membrane TRAIL relocalization and IFN-alpha production by human plasmacytoid dendritic cells in vitro and in vivo. *PLoS neglected tropical diseases* **7**, e2257, doi:10.1371/journal.pntd.0002257 (2013).

3 Kurane, I. *et al.* High levels of interferon alpha in the sera of children with dengue virus infection. *The American journal of tropical medicine and hygiene* **48**, 222-229 (1993).

4 Singla, M. *et al.* Immune Response to Dengue Virus Infection in Pediatric Patients in New Delhi, India--Association of Viremia, Inflammatory Mediators and Monocytes with Disease Severity. *PLoS neglected tropical diseases* **10**, e0004497, doi:10.1371/journal.pntd.0004497 (2016).

5 Dufour, J. H. *et al.* IFN-gamma-inducible protein 10 (IP-10; CXCL10)-deficient mice reveal a role for IP-10 in effector T cell generation and trafficking. *Journal of immunology (Baltimore, Md. : 1950)* **168**, 3195-3204 (2002).

6 Hsieh, M. F. *et al.* Both CXCR3 and CXCL10/IFN-inducible protein 10 are required for resistance to primary infection by dengue virus. *Journal of immunology (Baltimore, Md. : 1950)* **177**, 1855-1863 (2006).

7 Weiskopf, D. *et al.* Dengue virus infection elicits highly polarized CX3CR1+ cytotoxic CD4+ T cells associated with protective immunity. *Proceedings of the National Academy of Sciences of the United States of America* **112**, E4256-4263, doi:10.1073/pnas.1505956112 (2015).

8 Gagnon, S. J., Ennis, F. A. & Rothman, A. L. Bystander target cell lysis and cytokine production by dengue virus-specific human CD4(+) cytotoxic T-lymphocyte clones. *Journal of virology* **73**, 3623-3629 (1999).

9 George, J. *et al.* Prior Exposure to Zika Virus Significantly Enhances Peak Dengue-2 Viremia in Rhesus Macaques. *Scientific reports* **7**, 10498, doi:10.1038/s41598-017-10901-1 (2017).

10 Andrade, P. *et al.* Impact of pre-existing dengue immunity on human antibody and memory B cell responses to Zika. *Nature communications* **10**, 938, doi:10.1038/s41467-019-08845-3 (2019).

11 Priyamvada, L. *et al.* B Cell Responses during Secondary Dengue Virus Infection Are Dominated by Highly Cross-Reactive, Memory-Derived Plasmablasts. *J Virol* **90**, 5574-5585, doi:10.1128/jvi.03203-15 (2016).

12 Pantoja, P. *et al.* Zika virus pathogenesis in rhesus macaques is unaffected by pre-existing immunity to dengue virus. *Nature communications* **8**, 15674, doi:10.1038/ncomms15674 (2017).

13 Montoya, M. *et al.* Longitudinal Analysis of Antibody Cross-neutralization Following Zika Virus and Dengue Virus Infection in Asia and the Americas. *J Infect Dis* **218**, 536-545, doi:10.1093/infdis/jiy164 (2018).

14 Gallichotte, E. N. *et al.* Genetic Variation between Dengue Virus Type 4 Strains Impacts Human Antibody Binding and Neutralization. *Cell reports* **25**, 1214-1224, doi:10.1016/j.celrep.2018.10.006 (2018).

15 Collins, M. H. *et al.* Lack of Durable Cross-Neutralizing Antibodies Against Zika Virus from Dengue Virus Infection. *Emerging infectious diseases* **23**, 773-781, doi:10.3201/eid2305.161630 (2017).

16 Miller, J. D. *et al.* Human effector and memory CD8+ T cell responses to smallpox and yellow fever vaccines. *Immunity* **28**, 710-722, doi:10.1016/j.immuni.2008.02.020 (2008).

17 Knudsen, M. L. *et al.* Kinetic and phenotypic analysis of CD8+ T cell responses after priming with alphavirus replicons and homologous or heterologous booster immunizations. *Journal of virology* **88**, 12438-12451, doi:10.1128/jvi.02223-14 (2014).

18 Grifoni, A. *et al.* Prior Dengue virus exposure shapes T cell immunity to Zika virus in humans. *J Virol* **DOI 10.1128/jvi.01469-17**, doi:10.1128/jvi.01469-17 (2017).

19 Weiskopf, D. *et al.* Comprehensive analysis of dengue virus-specific responses supports an HLA-linked protective role for CD8+ T cells. *Proceedings of the National Academy of Sciences of the United States of America* **110**, E2046-2053, doi:10.1073/pnas.1305227110 (2013).

20 Mathew, A. *et al.* Predominance of HLA-restricted cytotoxic T-lymphocyte responses to serotype-cross-reactive epitopes on nonstructural proteins following natural secondary dengue virus infection. *Journal of virology* **72**, 3999-4004 (1998).

21 Hatch, S. *et al.* Intracellular cytokine production by dengue virus-specific T cells correlates with subclinical secondary infection. *J Infect Dis* **203**, 1282-1291, doi:10.1093/infdis/jir012 (2011).
