# Supplementary material for: Time elapsed between Zika and dengue virus infections affects antibody and T cell responses
Source: Nat Commun. 2019 Sep 20;10:4316. doi: 10.1038/s41467-019-12295-2 (PMC6754404; doi:10.1038/s41467-019-12295-2)
Supplement: Supplementary file 3 — Reporting Summary [file 41467_2019_12295_MOESM3_ESM.pdf]

nature research

Corresponding author(s): Carlos A. Sariol

Last updated by author(s): Aug 21, 2019

Reporting Summary

Nature Research wishes to improve the reproducibility of the work that we publish. This form provides structure for consistency and transparency in reporting. For further information on Nature Research policies, seeAuthors & Referees and theEditorial Policy Checklist .

Statistics

For all statistical analyses, confirm that the following items are present in the figure legend, table legend, main text, or Methods section.

n/a	Confirmed	
☐	☒	The exact sample size (*n*) for each experimental group/condition, given as a discrete number and unit of measurement
☐	☒	A statement on whether measurements were taken from distinct samples or whether the same sample was measured repeatedly
☐	☒	The statistical test(s) used AND whether they are one- or two-sided<br>*Only common tests should be described solely by name; describe more complex techniques in the Methods section.*
☒	☐	A description of all covariates tested
☐	☒	A description of any assumptions or corrections, such as tests of normality and adjustment for multiple comparisons
☐	☒	A full description of the statistical parameters including central tendency (e.g. means) or other basic estimates (e.g. regression coefficient) AND variation (e.g. standard deviation) or associated estimates of uncertainty (e.g. confidence intervals)
☐	☒	For null hypothesis testing, the test statistic (e.g. *F*, *t*, *r*) with confidence intervals, effect sizes, degrees of freedom and *P* value noted<br>*Give P values as exact values whenever suitable.*
☒	☐	For Bayesian analysis, information on the choice of priors and Markov chain Monte Carlo settings
☒	☐	For hierarchical and complex designs, identification of the appropriate level for tests and full reporting of outcomes
☒	☐	Estimates of effect sizes (e.g. Cohen's *d*, Pearson's *r*), indicating how they were calculated

Our web collection on statistics for biologists contains articles on many of the points above.

Software and code

Policy information about availability of computer code

Data collection	No software was used to collect samples.
Data analysis	GraphPad Prism version 7.0

For manuscripts utilizing custom algorithms or software that are central to the research but not yet described in published literature, software must be made available to editors/reviewers. We strongly encourage code deposition in a community repository (e.g. GitHub). See the Nature Research guidelines for submitting code & software for further information.

Data

Policy information about availability of data

All manuscripts must include a data availability statement. This statement should provide the following information, where applicable:

- Accession codes, unique identifiers, or web links for publicly available datasets
- A list of figures that have associated raw data
- A description of any restrictions on data availability

All relevant data is in main figures and supplementary information, any additional details are available from authors upon request. Source Data File included.

Field-specific reporting

Please select the one below that is the best fit for your research. If you are not sure, read the appropriate sections before making your selection.

☒ Life sciences ☐ Behavioural & social sciences ☐ Ecological, evolutionary & environmental sciences

For a reference copy of the document with all sections, see nature.com/documents/nr-reporting-summary-flat.pdf

Life sciences study design

All studies must disclose on these points even when the disclosure is negative.

Sample size	Sample size was determined by the guidelines published in the following reference: Sariol, C. A. et al., 2014 (doi:10.3389/fimmu.2014.00452), which use 4-6 macaques as a minimum to obtain accurate statistical power and to avoid genetic heterogeneity between animals. Also, we used as reference the standardization from the experimental design used in our last published work, Pantoja, P. et al., 2017 (doi:10.1038/ncomms15674). The sample size selected was sufficient for achieving statistical differences between ZIKV-immune groups and the ZIKV naive group, and even more between both ZIKV-immune groups.
Data exclusions	No data exclusions.
Replication	All replications were successful.
Randomization	No randomization was used for our study with an experimental animal model.
Blinding	No blinding was used for our study with an experimental animal model.

Reporting for specific materials, systems and methods

We require information from authors about some types of materials, experimental systems and methods used in many studies. Here, indicate whether each material, system or method listed is relevant to your study. If you are not sure if a list item applies to your research, read the appropriate section before selecting a response.

Materials & experimental systems

n/a	Involved in the study	
☐	☒	Antibodies
☐	☒	Eukaryotic cell lines
☒	☐	Palaeontology
☐	☒	Animals and other organisms
☒	☐	Human research participants
☒	☐	Clinical data

Methods

n/a	Involved in the study	
☒	☐	ChIP-seq
☐	☒	Flow cytometry
☒	☐	MRI-based neuroimaging

Antibodies

Antibodies used	Antibodies used for this study (Also detailed in supplementary information):<br>1. APC Mouse Anti-Human CD123 (clone 7G3) Obtained from BD-Biosciences (cat# 560087)<br>2. FITC Mouse Anti-Human CD20 (clone 2H7) Obtained from BD-Biosciences (cat# 555622)<br>3. FITC Mouse Anti-Human CD14 (clone M5E2) Obtained from BD-Biosciences (cat# 555397)<br>4. Alexa Fluor 700 Mouse Anti-Human CD16 (clone 3G8) Obtained from BD-Biosciences (cat# 555406)<br>5. BV421 Mouse Anti-Human CD20 (clone 2H7) Obtained from BD-Biosciences (cat# 302328)<br>6. PE Mouse Anti-Human CD69-PE (clone FN50) Obtained from BD-Biosciences (cat# 557050)<br>7. V500 Mouse Anti-Human CD14- (clone M5E2) Obtained from BD-Biosciences (cat# 561391)<br>8. FITC Mouse Anti-Ki-67 (clone B56) Obtained from BD-Biosciences (cat# 558616)<br>9. FITC Mouse Anti-Human CD3 (clone SP34) Obtained from BD-Biosciences (cat# 556611)<br>10. PerCP Mouse Anti-Human CD4 (clone M-T466) Obtained from Miltenyi Biotec (cat# 130-101-147)<br>11. Human Anti-HLA-DR-VioGreen (clone G46.6) Obtained from Miltenyi Biotec (cat# 130-111-795)<br>12. Human CD337 (NKp30)-PE-Vio770 (clone AF29-4D12) Obtained from Miltenyi Biotec (cat# 130-104-116)<br>13. Human CD8-VioGreen (clone BW135/80) Obtained from Miltenyi Biotec (cat# 130-096-902)<br>14. Human CD159a (NKG2A)-FITC (clone REA110) Obtained from Miltenyi Biotec (cat# 130-098-818)<br>15. Non-Human Primate CD3-PE-Vio770 (clone 10D12) Obtained from Miltenyi Biotec (cat# 130-104-202)<br>16. Human CD16-APC-Vio770 (clone VEP13) Obtained from Miltenyi Biotec (cat# 130-096-655)<br>17. Non-Human Primate CD3-APC (clone 10D12) Obtained from Miltenyi Biotec (cat# 130-091-998)<br>18. Human CD28-APC-Vio770 (clone 15E8) Obtained from Miltenyi Biotec (cat# 130-104-278)<br>19. Human CD56-PE (clone AF12-7H3) Obtained from Miltenyi Biotec (cat# 130-090-755)<br>20. Mouse Anti-Human CD335 (NKp46)-PC5 (clone BAB281) Obtained from Beckman-Coulter (cat# A66902)<br>21. PE/Cy7 anti-human CD11c (clone 3.9) Obtained from BioLegend (cat# 301608)<br>22. FITC Mouse Anti-Human CD8 (clone SK1) Obtained from BD (cat# 347313)<br>23. Anti-Human CD8-BV421 (clone SK1) Obtained from Biolegend (cat# 344748)<br>24. Anti-E mAb 4G2 provided by Dr. Aravinda de Silva (University of North Carolina, School of Medicine)<br>25. Anti-prM mAb 2H2 provided by Dr. Aravinda de Silva (University of North Carolina, School of Medicine)<br>26. Horseradish peroxidase (HRP)-conjugated goat anti-mouse antibody obtained from Sigma (cat# A3682)
Validation	The antibody panel used for immunophenotyping by flow cytometry (Antibodies 1-23) were validated for our previous work published in Nature Communications by Pantoja et al., 2017 (doi:10.1038/ncomms15674). Also, viral titrations by Plaque Assay and Plaque Reduction Neutralization Test (PRNT) were performed using antibodies 24-26 from the list as primary (24, 25), and conjugated secondary antibody (26) for this publication as well.

Eukaryotic cell lines

Policy information about cell lines

Cell line source(s)	Vero cells (ATCC CCL-81) are derived from kidney of african green monkeys (Cercopithecus aethiops). C6/36 cells (ATCC CRL-1660) are from Aedes albopictus larva.
Authentication	Cell lines authentication are provided by the manufacturer (ATCC).
Mycoplasma contamination	Contamination with Mycoplasma spp. was tested and no contamination was found.
Commonly misidentified lines<br>(See ICLAC register)	No misidentified cell lines were used in this study.

Animals and other organisms

Policy information about studies involving animals; ARRIVE guidelines recommended for reporting animal research

Laboratory animals	14 young adult male rhesus macaques (Macaca mulatta) matched in age and weight were housed and monitored in the Caribbean Primate Research Center (CPRC) and Animal Resources Center facilities, at the University of Puerto Rico-Medical Sciences Campus, San Juan, Puerto Rico.
Wild animals	No wild animals were used.
Field-collected samples	No field samples were collected for this study.
Ethics oversight	All the procedures were performed under the approval of the Institutional Animal Care and Use Committee (IACUC) of UPR-MSC and in a facility accredited by the Association for Assessment and Accreditation of Laboratory Animal Care (AAALAC file # 000593; Animal Welfare Assurance number A3421; protocol number, 7890116). Procedures involving animals were conducted in accordance with USDA Animal Welfare Regulations, the Guide for the Care and use of Laboratory Animals and institutional policies to ensure minimal suffering of animals during procedures.

Note that full information on the approval of the study protocol must also be provided in the manuscript.

Flow Cytometry

Plots

Confirm that:

- [x] The axis labels state the marker and fluorochrome used (e.g. CD4-FITC).
- [x] The axis scales are clearly visible. Include numbers along axes only for bottom left plot of group (a 'group' is an analysis of identical markers).
- [x] All plots are contour plots with outliers or pseudocolor plots.
- [x] A numerical value for number of cells or percentage (with statistics) is provided.

Methodology

Sample preparation	Aliquots of 150 ml of heparin whole blood were directly incubated with a mix of antibodies for 20 min. at RT. After incubation, RBCs were lysed, and cells were washed twice with PBS, followed by fixing with 1.6% methanol-free formaldehyde. Lysed and fixed samples were running in the flow cytometer.
Instrument	MACSQuant Analyzer 10 Flow Cytometer (Miltenyi Biotec, CA).
Software	FlowJo v.10
Cell population abundance	All cell subsets were determined from the processing of 150 ul of whole blood. Total PBMCs (100%) were quantified by flow cytometry and lymphocytes (25-40%) were gated. Total B (10-40%) and T (40-80%) cells were gated within total lymphocytes. The frequency, activation and proliferation of B cells and memory T cell subpopulations were determined and detailed in the supplementary information. The frequency of both main dendritic cells (DC) lineages (0.2-5%) within total PBMCs were also quantified. Also, the abundance of surface receptors in natural killer (NK) cell subpopulations were assessed as well. Detailed NK percentages are provided in a supplementary figure.
Gating strategy	Single cells (singlets) were selected by their FSC area (FSC-A) and height (FSC-H) patterns. Lymphocytes (LYM) were gated from singlets and based on their characteristic forward and side scatter pattern (FSC, SSC). T cells were selected gating on the CD3+ population. CD4+ and CD8+ T cells were defined as CD3+CD4+ and CD3+CD8+, respectively. Naive (N; CD28+CD95-), effector memory (EM; CD28-CD95+) and central memory (CM; CD28+CD95+) T cell subpopulations were determined within CD4+ and CD8+ T cells. B cells were defined as CD20+CD3-. The activation of B and T cell memory subpopulations (EM and CM) was assessed by the presence of the early activation marker CD69. Proliferation of total and activated B cells was quantified by the expression of the intracellular marker Ki67. Natural killer (NK) cells were defined as CD3-CD20-CD14- and analyzed by the expression of the following NK cell markers: CD8, CD56, NKG2A, NKp30, and NKp46. Dendritic cells (DC) were separated in two populations within the Lineage-DR+ (HLA-DR+ CD3- CD14- CD16- CD20- CD8- NKG2A-) by the expression of CD123 (plasmacytoid, pDC) or CD11c (myeloid, mDCs) and their percentages were calculated from total PBMCs.

- [x] Tick this box to confirm that a figure exemplifying the gating strategy is provided in the Supplementary Information.
